# A Single Sfp-Type Phosphopantetheinyl Transferase Plays a Major Role in the Biosynthesis of PKS and NRPS Derived Metabolites in *Streptomyces ambofaciens* ATCC23877

**DOI:** 10.1371/journal.pone.0087607

**Published:** 2014-01-31

**Authors:** Robert Bunet, Ramona Riclea, Luisa Laureti, Laurence Hôtel, Cédric Paris, Jean-Michel Girardet, Dieter Spiteller, Jeroen S. Dickschat, Pierre Leblond, Bertrand Aigle

**Affiliations:** 1 Université de Lorraine, Dynamique des Génomes et Adaptation Microbienne, UMR 1128, Vandœuvre-lès-Nancy, France; 2 INRA, Dynamique des Génomes et Adaptation Microbienne, UMR 1128, Vandœuvre-lès-Nancy, France; 3 Institute of Organic Chemistry, TU Braunschweig, Braunschweig, Germany; 4 Université de Lorraine, Laboratoire d’Ingénierie des Biomolécules, Ecole Nationale Supérieure d’Agronomie et des Industries Alimentaires, Vandœuvre-lès-Nancy, France; 5 Université de Lorraine, Unité de Recherche Animal et Fonctionnalités des Produits Animaux (URAFPA), Vandœuvre-lès-Nancy Cedex, France; 6 INRA,URAFPA, USC 340, Vandœuvre-lès-Nancy, France; 7 Department of Biology, Chemical Ecology/Biological Chemistry, University of Konstanz, Konstanz, Germany; University of Strathclyde, United Kingdom

## Abstract

The phosphopantetheinyl transferases (PPTases) are responsible for the activation of the carrier protein domains of the polyketide synthases (PKS), non ribosomal peptide synthases (NRPS) and fatty acid synthases (FAS). The analysis of the *Streptomyces ambofaciens* ATCC23877 genome has revealed the presence of four putative PPTase encoding genes. One of these genes appears to be essential and is likely involved in fatty acid biosynthesis. Two other PPTase genes, *samT0172* (*alpN*) and *samL0372*, are located within a type II PKS gene cluster responsible for the kinamycin production and an hybrid NRPS-PKS cluster involved in antimycin production, respectively, and their products were shown to be specifically involved in the biosynthesis of these secondary metabolites. Surprisingly, the fourth PPTase gene, which is not located within a secondary metabolite gene cluster, appears to play a pleiotropic role. Its product is likely involved in the activation of the acyl- and peptidyl-carrier protein domains within all the other PKS and NRPS complexes encoded by *S. ambofaciens*. Indeed, the deletion of this gene affects the production of the spiramycin and stambomycin macrolide antibiotics and of the grey spore pigment, all three being PKS-derived metabolites, as well as the production of the nonribosomally produced compounds, the hydroxamate siderophore coelichelin and the pyrrolamide antibiotic congocidine. In addition, this PPTase seems to act in concert with the product of *samL0372* to activate the ACP and/or PCP domains of the antimycin biosynthesis cluster which is also responsible for the production of volatile lactones.

## Introduction

Phosphospantetheinylation is absolutely required for the biosynthesis of fatty acids, polyketides and nonribosomally synthesized peptides [1]. This reaction, catalyzed by the Mg^2+^-dependent phosphopantetheinyl transferases (PPTases), allows the activation by posttranslational modification of the acyl carrier protein (ACP) domains in the fatty acid synthases (FASs) and polyketide synthases (PKSs) and of the peptidyl carrier protein (PCP) domains in the nonribosomal peptide synthases (NRPSs). This activation, which is essential for the function of the carrier protein domains, occurs by the transfer of the 4′-phosphopantetheine (*P*-pant) moiety of coenzyme A to a conserved serine residue within the active site of the protein substrates. The free thiol of the *P*-pant residue then permits attachment of the building blocks and the growing acyl or polypeptide chains to the carrier proteins as thioesters.

PPTases are divided into three groups based upon their sequence homologies and substrate spectra [2]. The members of the first group are usually associated with primary metabolism and catalyze the activation of the fatty acid ACPs. They have been classified as ACPS-type in reference to the ACPS (for holo-acyl carrier protein synthase) protein of *Escherichia coli*, the first *P*-pant transferase to be cloned and characterized [3]. PPTases of this group are usually about 120 aa in length and act as homotrimers. They have been shown to accept ACPs from type II PKSs as substrate *in vitro* [4] and probably *in vivo* [5]. The prototype of the second group of PPTases, Sfp, activates the PCP domains of the surfactin synthetase in *Bacillus subtilis* [6]. The Sfp-type PPTases are often approximately twice the size of the ACPS-type and they are monomeric in structure, but are considered as pseudo-dimeric because they are composed of two sub-domains resembling two ACPS monomers [7]. This group of enzymes exhibits broad spectrum activity. They can indeed modify PCP domains of NRPSs as well as ACP domains of PKSs and FASs. Nevertheless, the Sppt *P*-pant transferase of the cyanobacterium *Synechocystis sp*. PCC6803 is only able to activate its cognate fatty acid synthesis carrier protein [8]. Two distinct sub-families can be distinguished in the Sfp-type PPTases based on conserved amino-acid motifs [9], [10]: the F/KES family, which includes the majority of PPTases associated with NRPS and siderophore synthesis, and the W/KEA family which includes Sfp from *B. subtilis* and PPTases involved in polyketide biosynthesis as well as the enzymes in glycolipid or lysine biosynthesis. In bacteria, this type of PPTase genes has been found both in NRPS and PKS gene clusters [11], [12] but also independent of secondary metabolite gene clusters [13]. The third group of PPTases was identified in yeasts. They correspond to a domain of about 140 amino acids located in the C-terminal part of type I FAS that activates the ACP domain in *cis* by self-phosphopantetheinylation [14]. Such integrated PPTase domains were also identified in plants and within bacterial PKSs [9].

The bacteria belonging to the genus *Streptomyces* are well known for their ability to produce a wide range of natural products. Genome sequence analysis has revealed the presence of usually more than 20, sometimes 30, secondary metabolite gene clusters in these bacteria including numerous polyketides and non ribosomal peptides, many of which being used in human medicine. The genome of each *Streptomyces* species usually contains several PPTase genes, but their number does not correspond to the multiple *P*-pant requiring metabolic pathways. Indeed the number of ACP and PCP containing loci largely exceeds the number of PPTase genes as revealed by analysis of sequenced genomes. In the *Streptomyces* model, *Streptomyces coelicolor* A3(2), only three PPTases are present for 22 secondary metabolite gene clusters including two type II and type I PKS gene clusters, four NRPS gene clusters and the prodiginine producing *red* cluster which also encodes PCPs and ACPs [15], [16]. In *Streptomyces griseus*, the genome analysis has revealed two PPTases for no less than 16 clusters encoding PKS and/or NRPS [17], and in *Streptomyces avermitilis*, seven PPTase genes including a possible pseudogene and a domain within a type I PKS gene are present for 13 PKS and 8 NRPS gene clusters [18], [19]. This reflects the flexibility of the *P*-pant transferase proteins. However, a limited number of PPTase *in vivo* studies has been published for *Streptomyces* and to our knowledge none of them has studied in detail the set of the biosynthetic pathways under the dependency of a single PPTase. The reason is likely that most of the genomes have been just recently sequenced and that many metabolites produced by uncharacterized clusters are still unknown. The role of the three PPTase genes in *S. coelicolor* A3(2) was recently studied [5]. Among them, only *redU* (*sco5883*) is located within a secondary metabolite gene cluster, the *red* cluster responsible for the prodigiosin biosynthesis. RedU is only involved in prodigiosin production by activating the RedO PCP [5], [20]. The two other genes, *sco4744* and *sco6673*, encode an ACPS-type and an Sfp-type PPTase of the F/KES family. SCO4744 is likely responsible for the fatty acid biosynthesis: it is the only ACPS-type protein and no mutant for *sco4744* could be isolated as expected for an essential gene. But it is considered as a ‘promiscuous’ PPTase because the double mutant *redU*-*sco6673* was still able to synthesize actinorhodin (ACT) and the grey spore pigment, compounds produced by type II PKS gene clusters, and it has also been proposed to activate the ACP domains encoded within the *red* cluster [5]. On the other hand, the product of *sco6673* was shown to be required for the production of the non ribosomal lipopeptide, the calcium dependent antibiotic (CDA). It has been suggested that the SCO6673 PPTase could also participate in the synthesis of other non ribosomal peptides such as the siderophore coelichelin [5]. Thus, from this study, it is tempting to speculate that the PPTases SCO4744 and SCO6673 are involved in the activation of all ACPs and PCPs (with the exception of RedO) encoded within the *S. coelicolor* genome but their respective roles remain to be determined.

Before the genome sequence and despite decades of studies, *Streptomyces ambofaciens* ATCC23877 was only known to produce the cytotoxic pyrrolamide congocidine and the macrolide spiramycin, which is used in human medicine as antibacterial drug and for the treatment of toxoplasmosis. Mining of the *S. ambofaciens* ATCC23877 genome sequence has unveiled the presence of 25 secondary metabolite gene clusters [21]. The sequencing data also resulted in the full characterization of the spiramycin cluster, a type I PKS gene cluster [22] and the identification of the congocidine cluster, a NRPS gene cluster [23]. The analysis of cryptic clusters resulted in the discovery of several secondary metabolites produced by *S. ambofaciens* ATCC23877: the novel bioactive 51-membered macrolide stambomycins [24], the kinamycins [25], [26], the siderophores desferrioxamines E and B and coelichelin [27], the antifungal antimycins [28] and the antimycin-derived blastimycinones and related butenolides [29]. In fact, although *S. ambofaciens* ATCC23877 and *S. coelicolor* A3(2) are very close relatives [30], they share only 11 secondary metabolite genes or gene clusters (unpublished data), e.g. those coding for the desferrioxamine, coelichelin, geosmin and the grey spore pigment.

The *S. ambofaciens* ATCC23877 genome encodes four PPTase genes, two of them corresponding to the orthologues of *sco4744* and *sco6673* that will be designated *acpS*-like and *sco6673*-like, respectively. The two other genes are located within secondary metabolite gene clusters, *alpN* (*samT0172*) in the kinamycin type II PKS gene cluster [25] and *samL0372* in the antimycin hybrid NRPS-PKS gene cluster. No PPTase genes have been identified in the seven other clusters encoding PCP and/or ACP proteins/domains: a type II PKS cluster responsible for the spore pigmentation, three type I PKS clusters including the spiramycin and stambomycin loci, and three NRPS clusters including those responsible for the congocidine and coelichelin biosynthesis.

During the course of this work, the role of these *P*-pant transferases has been investigated, showing that the F/KES family member SCO66733-like has an unexpected large pleiotropic role in contrast to its orthologue in *S. coelicolor* A3(2).

## Materials and Methods

### Bacterial Strains, Plasmids and Growth Conditions

Bacterial strains, plasmids, BACs and cosmids used in this study are listed in Table 1. *Streptomyces* strains were manipulated as described previously [25], [31]. Morphological differentiation, in particular the ability to sporulate, was assessed on SFM medium [31]. Production of antibiotics was assessed on/in R2 (kinamycin and stambomycin) and on/in HT [31] or MP5 [32] (spiramycin, stambomycin and congocidine). Antimycin production was determined in SFM and the related volatile production, blastmycinones and butenolides, on SFM. Siderophore biosynthesis was assessed on R2YE agar plates [31]. *E. coli* strains were cultivated in LB liquid medium [33]. For λ *red* genes induction, 10 mM of filtered L-arabinose was added to the culture. *E. coli*, *Bacillus subtilis* ATCC6633 and *Micrococcus luteus* were used as indicator strains in the bioassays.

**Table 1:** List of strains, plasmids, cosmid and BACs used in this work.

Strains, BAC, cosmid or plasmid	Principal characteristics^a^	Source or Reference
*S. ambofaciens*ATCC23877	Wild-type (WT)	[48]
*S. ambofaciens*WT/pSET152	Empty vector pSET152 integrated in the *attB* site	This work
*S. ambofaciens*ATCC/OE484	Overexpression of the LAL regulator SAMR0484	[24]
*S. ambofaciens* ΔΔ*alpN*	The two copies of *alpN* in-frame deleted	This work
*S. ambofaciens* ΔΔ*alpN*/pSET152	Empty vector pSET152 integrated in the *attB* site	This work
*S. ambofaciens* ΔΔ*alpN*/pSET*alpN*	Mutant complemented with the *alpN* wt allele	This work
*S. ambofaciens* Δ*samL0372::apra-oriT*	The *samL0372* gene replaced by *the aac(3)-IV-oriT* mutagenesis cassette	This work
*S. ambofaciens* Δ*samL0372*	The *samL0372* gene in-frame-deleted	This work
*S. ambofaciens* Δ*sco6673-like*	The *sco6673-like* gene in-frame-deleted	This work
*S. ambofaciens* Δ*sco6673-like*/pIB139	Empty vector pIB139 integrated in the *attB* site	This work
*S. ambofaciens* Δ*sco6673-like*/pIB*sco6673-like*	Mutant complemented with the *sco6673-like* wt allele	This work
*S. ambofaciens* Δ*sco6673-like*/OE484	Overexpression of the LAL regulator SAMR0484	This work
*E. coli*DH5α	General cloning strain and strain used in bioassays	[49]
*E. coli* ET12567/pUZ8002	Nonmethylating strain with mobilization plasmid for conjugation with *Streptomyces*	[50]
*E. coli*BW25113/pKD20 or pIJ790	Strain used for the PCR-targeting mutagenesis (*gam*, *bet*, *exo*, *bla*)	[38] [35]
*B. subtilis* ATCC6663	Strain used as indicator in bioassays	
F6	Cosmid from the genomic library of *S. ambofaciens*(*neo*, *bla*)	[30]
F6Δ*alpN*::*aadA+oriT*	*alpN* replaced by *aadA-IV*/*oriT* cassette in F6 (*bla*, *neo*, spec)	This work
BAA13ZB5	BAC from the genomic library of *S. ambofaciens* (*cat*)	[30]
BAB6ZG10	BAC from the genomic library of *S. ambofaciens* (*cat*)	[30]
BAB29ZA2	BAC from the genomic library of *S. ambofaciens* (*cat*)	[30]
BAA13ZB5::*aadA*/Δ*acpS-like::aac(3)IV +oriT*	*acpS-like* replaced by an apramycin cassette and the *cat* gene bythe *aadA* gene (spec, apra)	This work
BAB6ZG10/Δ*samL0372::aac(3)IV +oriT*	*samL0372* replaced by an apramycin cassette (*cat*, apra)	This work
BAB29ZA2::*aadA/*Δ*sco6673-like::aac(3)IV+oriT*	*sco6673-like* replaced by an apramycin cassette and the *cat* gene bythe *aadA* gene (spec, apra)	This work
pIJ778	pBlusecript KS+, *aadA*+*oriT* cassette, FRT sites	[35]
pPSM88T	pOSV503 derivative containing *oriT*	[37] A. Thibessard, pers. com.
BT340	FLP recombination plasmid; *flpblacatrepA101*	[38]
pOSK1111	Conjugative plasmid with the *xis* and *int* genes of pSAM2	[37]
pGEMT-easy	PCR cloning vector, *bla*	Promega
pGEMT-*alpN*	*bla*, *alpN*	This work
pJET1.2/blunt	PCR cloning vector, *bla*	Thermo Scientific
pJET1.2-*sco6673-like*	*bla*, *sco-6673-like*	This work
pIB139	Conjugative and integrative plasmid (*oriT attP*_φC31_ *int*_φC31_ aac(3)IV ermEp*)	[51]
pSET152	Conjugative and integrative plasmid (*oriT attP*_φC31_ *int*_φC31_ *aac(3)IV*)	[52]
pIB-*sco6673-like*	pIB139+ *sco6673-like*	This work
pSET*alpN*	pSET152+ *alpN*	This work
pOE-0484	pIB139+ *samR0484*	[24]

a *bla*, ampicillin resistance gene; *neo*, kanamycin resistance gene; *aac(3)IV*, apramycin(apra) resistance gene; *oriT*, origin of transfer; *aadA*, spectinomycin/streptomycin (spec) resistance gene; *gam*, inhibitor of the host exonuclease V; *bet*, single-stranded DNA binding protein; *exo*, exonuclease promoting recombination along with *bet*; *cat*, chloramphenicol resistance gene; *attP*_φC31_, φC31 attachment site from the φC31 phage; *int*_φC31_, integrase gene of φC31; *flp*, FLP recombinase gene; *repA101*, thermosensitive replication origin; *xis* and *int*: excisionase and integrase of pSAM2, respectively.

### DNA Manipulation

Isolation, cloning, and manipulation of DNA were carried out as previously described in [31] for *Streptomyces* and in [33] for *E. coli*. Pulsed-field gel electrophoresis (PFGE) analyses were performed as previously described [34]. Amplification of DNA fragments by PCR was performed with *Taq* DNA polymerase (NEB) or Takara polymerase (Fermentas), according to the manufacturer’s instructions. When needed, PCR products and restriction fragments were purified from agarose gels with the High Pure PCR product purification kit (Roche).

### Construction of the *S. ambofaciens* Mutant Strains

The REDIRECT system [35] was used to make the in-frame deletion or gene replacement of the *S. ambofaciens* ATCC23877 PPTase encoding genes, as described [25], [36]. The *aadA*-*oriT* and *aac(3)-IV*-*oriT* mutagenesis cassettes used for gene replacement were synthesized by PCR using pIJ778 [35] and pSPM88T (Annabelle Thibessard, pers. com.) [37] as templates, respectively. *E. coli* BW251113/pKD20 [38] was first transformed with the BAC containing the PPTase gene of interest (*samL0372*, *sco6673-like* or *sco4744-like*), and then with the PCR product (*aac(3)-IV*-*oriT* mutagenesis cassette) to replace the targeted gene by homologous recombination. For the deletion of the two copies of *alpN*, *E. coli* BW251113/pIJ790 [35] was used instead of *E. coli* BW251113/pKD20 and was transformed with the F6 cosmid and then with the *aadA*-*oriT* cassette. The chloramphenicol resistance gene of the vector pBelo-BAC11 was replaced by a spectinomycin resistance gene, using the same strategy. *E. coli* ET12567/pUZ8002 was transformed with the mutated BACs or cosmid for conjugation with *S. ambofaciens* ATCC23877. Gene replacements were confirmed by PCR analysis using primer sets flanking the targeted genes and/or Southern blot analyses. PFGE analysis was also carried out for the *alpN* mutants to rule out the formation of large genomic rearrangements. To get in frame deleted mutants of *alpN* and of *samL0372* and *sco6673-like*, the cassette was removed using respectively the Flip recombinase as described in [35] and the excisionase and integrase of pSAM2 as described in [37]. Only the start and stop codons of the genes remained after deletion. All primers used to generate the cassettes and to confirm the gene deletion are described in Table S1. For each gene, at least three independent mutants were isolated and analyzed for the secondary metabolite production with the exception of *alpN* for which two independent mutants were studied.

### Complementation of the PPTase Mutants

To complement the ΔΔ*alpN* mutants, a strategy similar to the one already used for other *alp* genes was used [39]. Briefly, the *alpN* gene including its putative promoter was amplified from the F6 cosmid with the primer set alpN-fwd/alpN-prom using high-fidelity Phusion polymerase (Finnzyme). After a standard A-tailing step, the 1,158-bp PCR product was cloned into pGEMT-easy (Promega) and the integrity of the insert was confirmed by sequencing. After restriction by *Eco*RI, the insert was cloned into pSET152 previously digested with the same enzyme to give pSET-*alpN* which was introduced in *S. ambofaciens* ATCC23877 by conjugation from *E. coli* (the plasmid integrates into the *Streptomyces* chromosomal φC31 attachment site by site-specific recombination). For the complementation of the Δ*sco6673-like* mutants, the strategy was slightly different since the 5′ end of the gene overlaps with the 3′ end of the upstream gene. Therefore, only the orf of *sco6673-like* was amplified from the *S. ambofaciens* ATCC23877 genomic DNA with the Phusion high-fidelity polymerase (Thermo Scientific). The PCR product was cloned into pJET1.2/blunt and the integrity of the insert was confirmed by sequencing. After restriction digestion with *Nde*I and *Xba*I, the insert was cloned into the conjugative and integrative pIB139 vector under the control of the ermEp* promoter modified to have a typical *Streptomyces* ribosome binding site [36] giving the pIB-*sco6673-like* plasmid. Empty vectors pSET152 and pIB139 were used as controls.

### Bioassays

To detect the production of kinamycins, bioassays were carried out from a plug of *S. ambofaciens* clones grown on R2 agar as previously described [25] using *B. subtilis* ATCC6633 as indicator strain. The congocidine bioassays were carried out on HT solid medium as described in [23] using *E. coli* DH5α as indicator strain.

### Reverse-phase HPLC, LC-MS and GC-MS Analyses

The production of spiramycins and congocidine was assessed from MP5 liquid grown cultures of *S. ambofaciens*. After 4 days cultivation at 30°C, supernatants were filtered through Phenex-RC membrane (0.45 µm; Phenomenex) and 100 µl were analyzed by RP-HPLC on an Alliance HPLC unit equipped with a photodiode array detector 996 (Waters, Milford, USA) and with a Lichrosphere RP18 column (150×2 mm, 5 µm particle size and 10 nm porosity; Merck). A linear gradient from 5% to 75% acetonitrile in water was applied in the presence of 0.1% of trifluoroacetic acid for 70 min with a flow rate of 0.25 ml/min at a temperature of 30°C. Absorption was monitored at 232 nm for spiramycins and congocidin and at 297 nm specifically for congocidine. Purified spiramycins and congocidine were used as standard. The presence of stambomycins was determined from the *S. ambofaciens* wt and mutant strains overexpressing *samR0484* (this gene lies within the stambomycin cluster and encodes a pathway-specific regulator of the LAL family and its overexpression is required to trigger the transcription of the stambomycin biosynthetic genes; [24]). LC-MS analyses were carried out from methanol extracts of mycelia grown either in MP5 liquid medium at 30°C [24] or on cellophane membranes lifted on R2 agar plates (4 days at 30°C). In the latter case, LC-MS analyses were carried out on an LTQ (ThermoFisher Scientific) ion trap mass spectrometer (Text S1). For antimycin detection, the strains were cultivated in SFM liquid medium at 28°C for three days and then culture samples (2 ml) were withdrawn, lyophylized, resuspended in 1 ml methanol and analyzed by LC-MS [28]. The production of blastmycinones and butenolides was assessed from cultures of *S. ambofaciens* strains grown on SFM plates for three to five days at 28°C. The volatiles were collected by use of a closed loop stripping apparatus as previously described [40] and GC-MS analyses of headspace extracts were performed as in [29]. The production of the siderophore coelichelin and desferrioxamines B and E was assessed from a *S. ambofaciens* culture grown on R2YE agar as described elsewhere [41]. Briefly, after growth on cellophane lifted on agar plates during 4 days at 30°C, siderophores were extracted from spent agar with one volume of MilliQ water, and, after lyophylisation, resuspended in MilliQ water according to the measured fresh weight of the biomass. The extracted siderophores were then analyzed by LC-ESI-MS (ThermoFisher Scientific) as in [41] (see Text S1).

## Results

### An Essential Role for the *acpS*-like Gene

The *acpS*-like gene of *S. ambofaciens* ATCC23877 is located within a region that is highly synthenic with the *S. coelicolor* region containing *sco4744*. Its product (123 aa) is homologous to PPTases of the ACPS family (Figure S1) and it shares 91% identity (95% similarity) with SCO4744. Similarly to *sco4744* [5], we could not obtain any mutant of this gene. Numerous attempts of gene replacement were unsuccessful and only clones with a single crossover were obtained. Therefore, the *acpS*-like gene is likely essential and may be involved in fatty acid biosynthesis as proposed for its orthologue in *S. coelicolor* A3(2). Nevertheless, contrary to SCO4744, the ACPS-like PPTase does not seem to be capable of *in vivo* activation of ACPs involved in polyketide synthesis (see below).

### AlpN, a PPTase Dedicated to Kinamycin Biosynthesis

The *samT0172* gene (also designated *alpN*) is present in two copies on the *S. ambofaciens* ATCC23877 chromosome since it is part of the duplicated type II PKS gene cluster responsible for the kinamycin production [25], [26]. Its product (269 aa) is a Sfp-type PPTase that belongs to the W/KEA subfamily. AlpN shares the highest similarity with SSDG_05480 (70% identity/79% similarity) and ORF56 (60%/68%) which are encoded by *Streptomyces pristinaespiralis* (NCBI Reference Sequence: ZP_06913971.1) and within a type I PKS gene cluster located on the linear plasmid pSLA2-L of *Streptomyces rochei* [42] (Fig. S2), respectively. It has no orthologue in *S. coelicolor* A3(2) and shows only a weak identity (31%) with RedU which is involved in the prodigiosin biosynthesis and is also member of the W/KEA subfamily.

**Figure 1:**
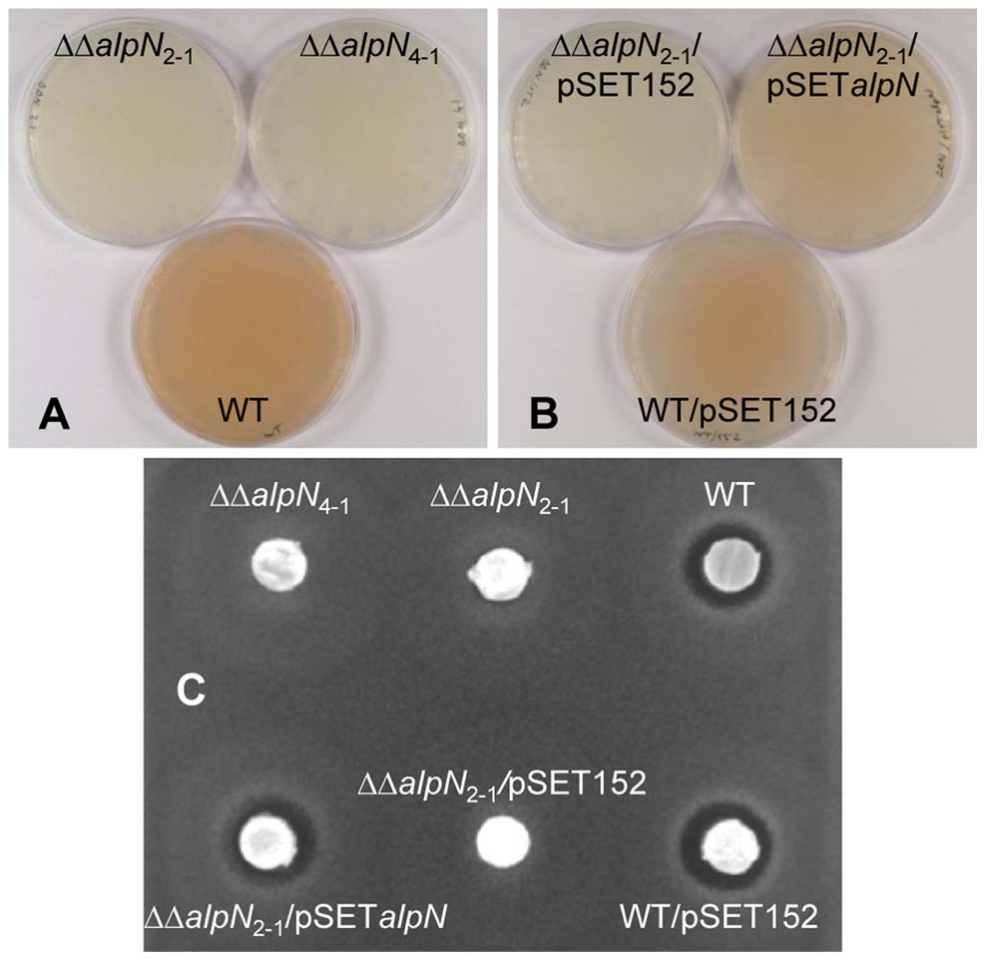
Effect of *alpN* deletion on production of the diffusible orange pigment and kinamycin. (A) Pigment synthesis was assessed on R2 plates in the wild-type (WT) strain and *alpN* double deletion mutants (two independent clones ΔΔ*alpN*_2-1_ and ΔΔ*alpN*_4-1_ are shown) and (B) in the ΔΔ*alpN*_2-1_ mutant carrying the pSET152 derivative pSET*alpN* in comparison with the WT/pSET152 and ΔΔ*alpN*_2-1_/pSET152 control strains. The photos were taken from below the plate. (C) Kinamycin production was visualized by the inhibition of *B. subtilis* growth. *Streptomyces* strains were grown on R2 agar, and a plug of mycelia was placed on an LB plate seeded with *B. subtilis*.

Based on its location within the *alp* cluster, the *alpN* product may activate the unique ACP (AlpC) encoded within the *alp* cluster [25]. To confirm this hypothesis, the two copies of the *alpN* open reading frames were removed by in-frame deletion. The mutants, designated ΔΔ*alpN*, showed growth and morphological differentiation identical to those of the wt strain under different growth conditions (data not shown). In particular, the grey color characteristic of mature spores was visible indicating that AlpN is not responsible for the activation of the carrier protein involved in the spore pigment biosynthesis. The *alp* cluster genes were previously shown to be associated with the production of kinamycin and a diffusible orange pigment on solid or in liquid R2 medium, the pigment being likely either a degradation or modification product of kinamycin [25], [26]. On R2 surface-grown cultures, no pigment could be detected in the ΔΔ*alpN* mutant strain after an incubation time of up to seven days, while orange pigmentation was clearly visible after 24 hours of growth in the parental strain (Fig. 1A). Plugs from agar plates were then assessed for their ability to inhibit the growth of *B. subtilis*, which is sensitive to kinamycins (Fig. 1C). Only the *S. ambofaciens* wt strain was active against the indicator strain. The reintroduction of a copy of *alpN* under the control of its own promoter in the ΔΔ*alpN* clones by using the plasmid pSET*alpN* restored both pigment and antibiotic production (Fig. 1B; Fig. 1C), thus confirming the direct link between the deletion of the two copies of *alpN* and the phenotypes observed in the mutants.

Since the type I PKS gene clusters responsible for the production of the spiramycin and stambomycin macrolides do not contain a PPTase gene, we next checked if the deletion of *alpN* impaired the biosynthesis of these compounds. HPLC analysis of the culture supernatant of the ΔΔ*alpN* clone grown in MP5, a suitable medium for the macrolide production, showed that the deletion of this gene did not affect spiramycin production (Fig. S3). The effect on stambomycin biosynthesis was determined with the ΔΔ*alpN* clone overexpressing *samR0484* (ΔΔ*alpN*/OE484; see Materials and Methods). Analysis of the mycelium extract of ΔΔ*alpN*/OE484 by LC-MS also revealed that the biosynthesis of stambomycins is not impaired in the mutant (Fig. S4).

In a similar way, blastmycinones and butenolides, the volatiles that are degradation products of the antimycins, were detected in the ΔΔ*alpN* mutant (Fig. S5). Altogether, these data strongly suggest that AlpN is only required for the activation of the AlpC ACP involved in the biosynthesis of the aromatic polyketide kinamycin.

### SAML0372, an Unusual PPTase

**Figure 2:**
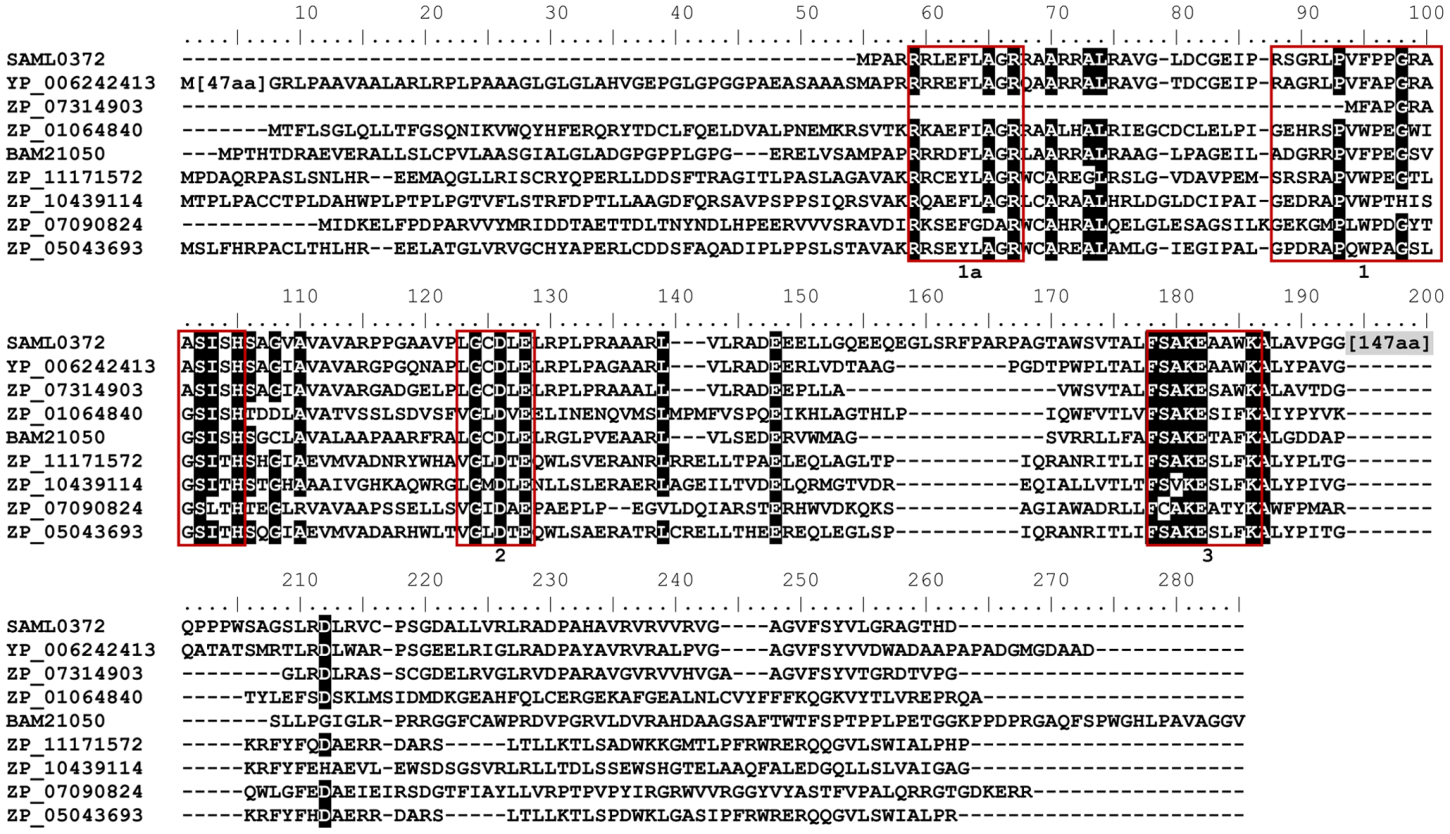
Sequence alignment of SAML0372 of *S. ambofaciens* ATCC23877 with other Sfp-type PPTases. The aa residues conserved in at least 7/9 proteins are shaded in black. SAML0372 belongs to the F/KES subfamily and the motifs characteristic of this subfamily [9] are red boxed. YP_006242413: *Streptomyces hygroscopicus subsp. jinggangensis* 5008; ZP_07314903: *Streptomyces griseoflavus* Tü4000; ZP_01064840: *Vibrio sp.* MED222; BAM21050: *Streptomyces blastmyceticus*; ZP_11171572: *Alcanivorax hongdengensis* A-11-3; ZP_10439114: *Pseudomonas extremaustralis* 14-3 *substr*. 14-3b; ZP_07090824: *Corynebacterium genitalium* ATCC 33030; ZP_05043693: *Alcanivorax sp.* DG881.

The hybrid NRPS-PKS gene cluster responsible for the biosynthesis of antimycins and of the related volatile compounds blastmycinones and butenolides [28], [29], is the other cluster encoding a PPTase (SAML0372). Surprisingly, while the three characterized antimycin clusters are well conserved (those identified in *Streptomyces albus* J1074, *Streptomyces* S4 and *S. ambofaciens* ATCC23877 [43]), *samL0372* as well as the downstream small orf *samL0373* of unknown function are present only in *S. ambofaciens* in the middle of the cluster. In addition, SAML0372 is a protein of 337 aa that contains a long spacer (147 aa) which is absent in all other PPTases characterized so far (Fig. 2). We nevertheless considered SAML0372 as a potential PPTase because it contains the three amino acid motifs characteristic of a PPTase, and also the residues participating in substrate binding and catalysis are present in the protein (Fig. 2). Although SAML0372 contains a FxxKEA domain, it was assigned to the F/KES subfamily since it possesses in addition to the three motifs conserved within PPTases the motif 1a specific to this subfamily [9].

**Figure 3:**
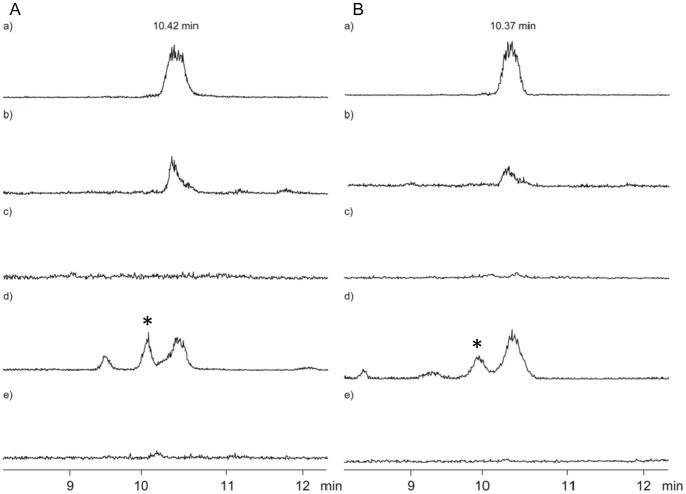
Effect of the deletion of *samL0372* and *sco6673-like* on the antimycin production. (A) LC-MS ion chromatograms (*m*/*z* = 571, [M+Na]^+^) of a) antimycin A_1_ and of methanolic culture extract of the *S. ambofaciens* ATCC23877 b) wild-type, c) Δ*sco6673-like*, d) Δ*sco6673-like*/pIB*sco6673-like* and e) Δ*samL0372* stains grown in liquid SFM. (B) LC-MS ion chromatograms (*m*/*z* = 557, [M+Na]^+^) of a) antimycin A_2_ and of methanolic culture extract of the *S. ambofaciens* ATCC23877 b) wild-type, c) Δ*sco6673-like*, d) Δ*sco6673-like*/pIB*sco6673-like* and e) Δ*samL0372* strains grown in liquid SFM. The peaks marked by an asterisk represent an unidentified compound that is not from the antimycin family.

To determine the role of this atypical PPTase, independent mutants deleted for s*amL0372* were isolated. Like the ΔΔ*alpN* strains, the *samL0372* mutants were not affected in growth and morphological differentiation including grey spore pigment production (data not shown). The production of antimycins and its degradation products, the volatile blastmycinones and butenolides, were surveyed by LC-MS and by use of a closed-loop stripping apparatus (CLSA) in combination with GC-MS, respectively. The mutant strains did not produce the antimycins A1 to A4 that are present in the extract of the wt strain (Fig. 3). Accordingly, GC-MS analysis confirmed the absence of blastmycinones and butenolides in the volatile fraction emitted by the agar plate culture of the mutants, while these compounds were produced in large amounts by the parental strain under the same growth conditions (Fig. S5). The dilactone scaffold of the antimycins is generated by a hybrid NRPS-PKS assembly line which involves a PCP protein, two PCP domains within the dimodule NRPS and an ACP domain within the PKS [44]. Therefore, our results strongly suggest that SAML0372 activates either the acyl- or peptidyl carrier domains or both involved in the antimycin production. Nevertheless, the analysis of the *sco6673-like* mutant (see below) indicates that SAML0372 probably acts only on one type of domain (ACP or PCP), but not on both.

Based on its location within the antimycin cluster, we suspected that the role of the *samL0372* gene product was specifically dedicated to the production of the antimycins and of the related volatiles. Indeed, when grown on R2 agar plates, the mutant strains still produced the orange pigment and showed antibacterial activity against *B. subtilis* likely due to the kinamycin production (Fig. S6). In addition, on HT or MP5 agar plates, the mutant strains exhibited antibacterial activity against *E. coli* indicating that the mutant was still able to produce congocidine (the other known antimicrobial compounds produced by *S. ambofaciens* ATCC23877 are not active against *E. coli*), as confirmed by HPLC analysis of liquid culture extracts (Fig. S6). Finally, HPLC analysis of MP5 liquid culture extracts confirmed that the production of spiramycin was not impaired in the mutant strain (Fig. S6). Therefore, the role of SAML0372 is likely limited to the antimycin biosynthetic pathway.

### A Pleiotropic Role for the Product of *sco6673-like*

Based on the roles identified for the other PPTases, it appears that the *sco6673-like* gene could play a pleiotropic role and may be involved in multiple *P*-pant requiring pathways in which the other PPTases do not act. Therefore, its role could be different to the one described for its orthologue *sco6673* in *S. coelicolor* A3(2). Its product (226 aa) shows 83% identity (87% similarity) with the SCO6673 protein of *S. coelicolor* A3(2) and belongs to the F/KES subfamily (Fig. S7).

**Figure 4:**
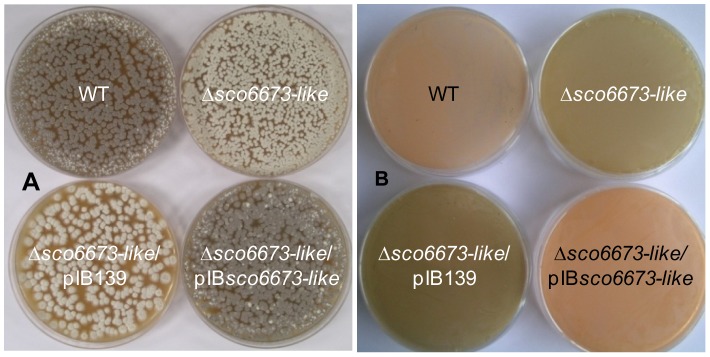
Effect of the deletion of *sco6673-like* on the spore pigment and orange pigment production. (A) Pigmentation of the *S. ambofaciens* colonies grown on SFM agar plates after 6 days at 30°C. The Δ*sco6673-like* mutant containing or not the vector is whitish while the complemented mutant strain (Δ*sco6673-like*/pIB*sco6673-like*) shows a grey pigmentation similar to the WT strain; (B) Orange pigment synthesis was assessed on R2 plates in the wild-type (WT) strain and in the Δ*sco6673-like* mutant as well as in the complemented Δ*sco6673-like* mutant (Δ*sco6673-like*/pIB*sco6673-like*) and the control strain Δ*sco6673-like*/pIB139. The photo was taken from below the plate.

Contrary to the other PPTase mutants, the deletion of *sco6673-like* appears to affect the morphological differentiation. Indeed, when grown on agar plates (SFM agar medium), the mutant colonies did not show the characteristic spore pigmentation (Fig. 4A) suggesting that the protein SCO6673-like is responsible for the phosphopantetheinylation of the ACP involved in the biosynthesis of the spore pigment. Complementation experiments with the pIB-*sco6673-like* plasmid (see Material and Methods) confirmed the involvement of SCO6673-like in the spore pigment production (Fig. 4A). It should be noted that the *sco6673-like* gene probably forms a single transcriptional unit with the upstream and overlapping gene which codes for a protein of unknown function but for the complementation only *sco6673-like* was placed downstream of ermE*p. Analysis of the colonies by scanning electron microscopy revealed that the deletion of *sco6673-like* does not seem to impair the ability of the mutant to sporulate (Fig. S8). Therefore, these results support the involvement of *sco6673-like* in the spore pigment biosynthesis.

**Figure 5:**
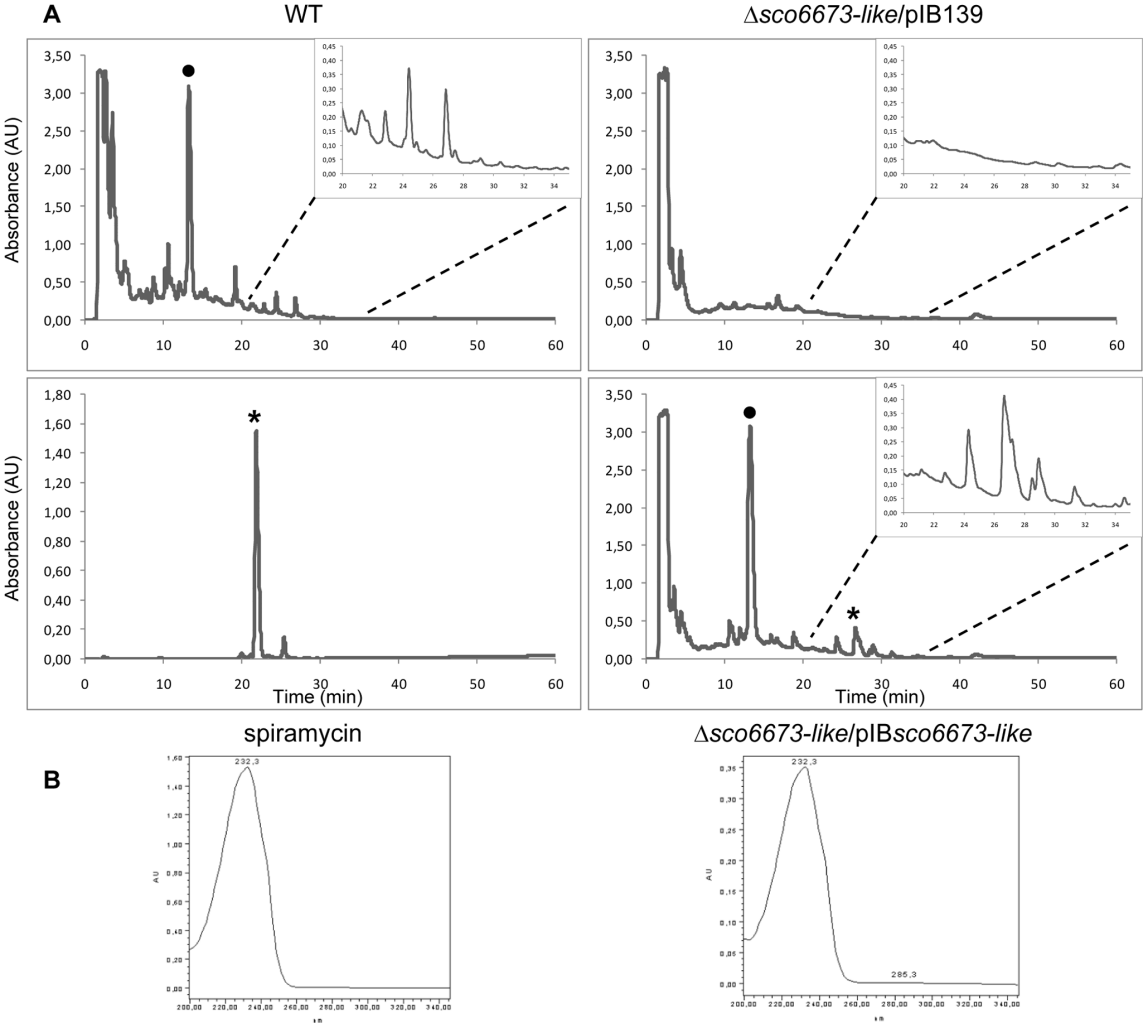
Effect of the deletion of *sco6673-like* on the spiramycin and congocidine production. (A) Spiramycin and congocidine production was assessed directly by HPLC from supernatant samples collected from the culture of *S. ambofaciens* ATCC23877 wild-type and mutant strains grown in liquid MP5. Commercial spiramycin (100 µl at 0.1 mg/ml) was used as control. Absorption was monitored at 232 nm. The inserts are an enlargement of the area between 20 to 35 min of retention time containing the peaks corresponding to the spiramycin (spiramycin is a mixture of three forms). The peak corresponding to congocidine is highlighted with a black dot. (B) UV spectra (from 200 to 350 nm) of the peaks highlighted with an asterisk and corresponding to spiramycin.

We then analyzed the biosynthesis of the secondary metabolites known to depend on clusters devoid of any PPTase gene. Bioassays and HPLC analysis of culture extracts were carried out from strains grown on MP5 and HT agar plates, since these media are known to be suitable for the production of spiramycin and congocidine. The Δ*sco6673-like*::scar mutant did not exhibit any activity onto the congocidine sensitive *E. coli* compared to the wt strain. Complementation experiments (strain Δ*sco6673-*like/pIB-*sco6673-like*) confirmed that the deletion of *sco6673-like* was responsible for the loss of the antibacterial activity (Fig. S9) and HPLC analyses of supernatant extracts from liquid cultures grown in MP5 verified that the deletion of *sco6673-like* in *S. ambofaciens* ATCC23877 prevents the production of congocidine (Fig. 5). Analogous results were obtained for the spiramycin production (Fig. 5).

**Figure 6:**
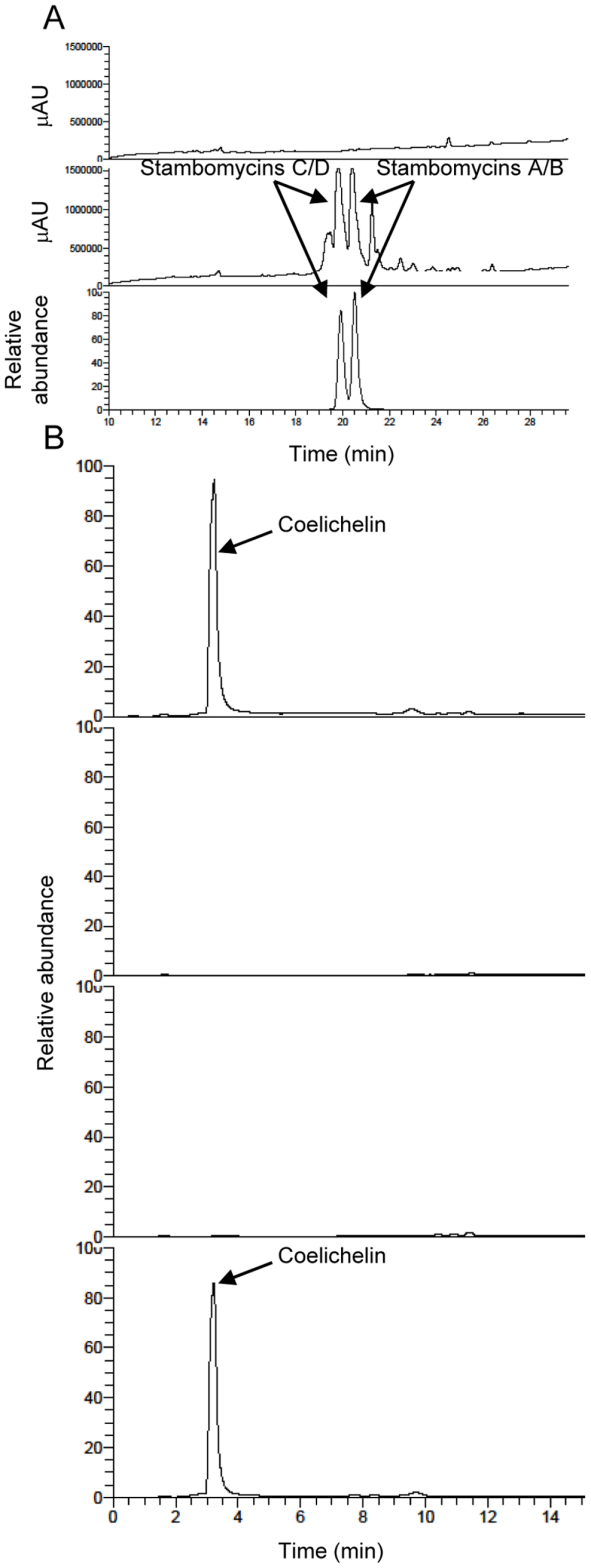
Effect of the deletion of *sco6673-like* on the stambomycin and coelichelin production. (A) Stambomycin production: UV chromatograms at 215 nm from LC-MS analyses of the extracts of Δ*sco6673-like*/OE484 (top) and ATCC/OE484 (middle) and ion chromatogram (bottom, m/z 682 and 689) corresponding to the dicharged [M+2H]^2+^ stambomycins C/D (m/z 682) and stambomycins A/B (m/z 689) [24]. (B) Coelichelin production: ion chromatograms from LC-MS analyses of the extracts, from top to bottom, of the *S. ambofaciens* wild type strain, the Δ*sco6673-like* mutant, the Δ*sco6673-like*/pIB139 strain and the Δ*sco6673-like*/pIB*sco6673-like* strain. Peaks with a retention time of 3.2 min correspond to the desferri- form of coelichelin.

The production of stambomycins in the mutant strain was tested from a clone overexpressing *samR0484* (strain Δ*sco6673-like*/OE484) and grown on R2 agar medium. LC-MS analyses from methanolic mycelium extracts showed that the macrolides are not detectable in the mutant extracts while the wt strain carrying pOE-0484 (ATCC/OE484) produced large amount of stambomycins (Fig. 6; Fig. S10). These data demonstrated that the acyl-carrier domains of the stambomycin biosynthetic pathways are activated by the PPTase SCO6673-like.

The last known compound whose production was hypothesized to be dependent of SCO6673-like is coelichelin. Recently, it has been shown that the siderophores coelichelin and desferrioxamines are produced by *S. coelicolor* A3(2) on R2YE solid medium [41]. From the same growth conditions, LC-MS analyses revealed the presence of coelichelin only in the wt strain but not in the Δ*sco6673-like*::scar mutant (Fig. 6; Fig. S10). The production of this molecule was fully restored in the complemented mutant. Thus, we concluded that the PPTase SCO6673-like activates the PCP domains of the NRPS required for coelichelin biosynthesis. The LC-MS analyses also showed that the production of the desferrioxamine E and B was not impaired by the deletion of *sco6673-like* (data not shown), as expected, since they are members of the nonpeptide hydroxamate siderophores.

Although SCO6673-like was not expected to be involved in the kinamycin and antimycin pathways, we nevertheless analyzed the mutant strain for the production of these metabolites. Surprisingly, on R2 medium, a diffusible brown pigmentation was observed around the mutants instead of the orange pigment observed with the parental strain (Fig. 4B). This phenotype was directly linked to the deletion of *sco6673-like* since the reintroduction of the wt allele in the mutant restored the orange pigmentation (Fig. 4B). We hypothesize that the level of kinamycin and pigment production is higher in the mutant strain compared to the wt strain due to a larger amount of available precursors in the mutant. Unexpectedly, the production of antimycins was abolished in the Δ*sco6673-like* mutant grown on SFM agar medium as observed by LC-MS analysis (Fig. 3). Complementation with the wt allele of *sco6673-like* restored the production (Fig. 3). These data revealed that the product of *sco6673-like* acts in concert with SAML0372 for the activation of the carrier domains involved in the biosynthesis of the antifungal compounds. However, the targets of SAML0372 and SCO6673-like have not been identified. Nevertheless, they should be different since the absence of one of the PPTase was not counterbalanced by the presence of the other. Therefore, one may speculate that one of these PPTases may activate the ACP domains while the other could act on the PCP domains encoded within the antimycin biosynthetic gene cluster.

## Discussion

We have determined the role of the four PPTase genes encoded within the genome of *S. ambofaciens* ATCC23877. The only PPTase of the ACPS-type family, SCO4744-like, is likely responsible for the activation of the carrier protein involved in the fatty acid biosynthesis since its gene appears to be essential, as its orthologue in *S. coelicolor* A3(2) [5]. Therefore, although the bacteria of the *Streptomyces* genus encode several Sfp-type genes, their products cannot complement the activity of ACPSs, a situation different than the one observed for the promiscuous Sfp of *B. subtilis* [45]. In addition, the role of the *Streptomyces* ACPS seems to vary from one species to another. Thus, in *S. ambofaciens*, the role of this transferase is more restricted than in *S. coelicolor* in which SCO4744 was reported to be also competent *in vivo* for the modification of ACPs involved in the actinorhodin, undecylprodigiosin and spore pigment production [5]. Recently, it has been reported that the ACPS-like enzyme of *Streptomyces chattanoogensis* L10 was also required for the spore pigment production in addition to its involvement in the fatty acid production [46]. This suggests that the function of two related genes/proteins has evolved differently in the two closely related *Streptomyces* species. A similar situation has been encountered with the Sfp-type PPTases, SCO6673-like of *S. ambofaciens* and SCO6673 of *S. coelicolor*. While in *S. coelicolor* the latter PPTase has been proposed to activate specifically PCPs [5], as usually described for the PPTases of the F/KES family, its role appears to be promiscuous in *S. ambofaciens*: it not only modifies PCPs (e.g. the one involved in the siderophore coelichelin production), but also ACPs (e.g. the ACP involved in the spore pigment biosynthesis or the carrier domains of the type I PKSs responsible for the spiramycin production). SCO6673-like is nevertheless not active on all ACPs, since AlpC, encoded by the type II PKS *alp* cluster (kinamycin) is transformed into its holo form by the Sfp-type AlpN, a member of the W/KEA family. At this stage, it is difficult to explain the difference of specificity between SCO6673 and SCO6673-like. The proteins are highly conserved (83%/87% of identity/similarity at the aa level) and the residues essential for the activity and structural stability within the conserved motifs (Fig. S7) are shared by the two enzymes. In addition, they show the same genetic organisation, *i.e.* like in *S. coelicolor* A3(2) (and the majority of the other *Streptomyces* sequenced genomes) the PPTase gene is located downstream of and overlapping a conserved gene of unknown function with a calcineurin-like phosphoesterase domain (Pfam00149). Some residues that are different between the two PPTases (e.g. those located within the conserved motifs) might explain the specificity of each of the enzymes, or additional factors might influence the specificity of the PPTases (such as the product of the upstream gene). It will be interesting to test if the SCO6673 protein of *S. coelicolor* A3(2) can substitute its orthologue in *S. ambofaciens*. To the best of our knowledge, it is the first time that, in *Streptomyces*, a Sfp-type protein is demonstrated *in vivo* to possess such a relaxed specificity towards a wide range of partners including both peptidyl- and acyl-carrier proteins/domains. Only the ACPs involved in kinamycin and fatty acid biosynthesis and probably some of the carrier proteins or domains (PCP or ACP) involved in antimycin production show no activation by SCO6673-like (see below). In addition, SCO6673-like could be involved in the activation of the ACP and PCP domains encoded by the still cryptic type I PKS and NRPS gene clusters identified in the *S. ambofaciens* ATCC23877 chromosome by genome mining. Other members of the F/KES family in *Streptomyces* are also flexible in terms of carrier proteins such as KirP of *Streptomyces collinus* Tü365 which targets ACP and PCP domains of the kirromycin NRPS/PKS [47], but none of them have been reported to activate the two types of carrier proteins from different secondary metabolite biosynthetic pathways *in vivo*.

As expected, the products of the genes located within secondary metabolite gene clusters are specifically dedicated to the production of their related compounds. Thus, our data show that AlpN is involved only in the production of the kinamycin antibiotics and of its related orange pigment. SAML0372 is necessary in the biosynthesis of the antifungal antimycins and of the blastmycinones and butenolides. As described above, SAML0372 appears to be a very unusual PPTase with a long spacer located between the motif 3 and the “classical” C-terminal part of the Sfp-type PPTases of *Streptomyces*. No PPTase with such a characteristic structure could be detected in protein databases. Only another strain of *S. ambofaciens*, the strain DSM40697 encodes an orthologue of *samL0372* also within an antimycin biosynthetic gene cluster (data not shown). In addition, no gene encoding a PPTase is present in the antimycin gene clusters of *S. albus* J1074 and *Streptomyces* S4 [43]. Similarly, *samL0373*, the gene immediately downstream *samL0372*, is also absent in these clusters but present in the *S. ambofaciens* DSM40697 strain. This suggests that the ancestor of the *S. ambofaciens* strains could have acquired this locus of two genes by a horizontal gene transfer event. The PPTase SAML0372 may have evolved to activate either the acyl or peptidyl carrier protein/domains involved in the biosynthesis of antimycins (and consequently of the blastmycinones and butenolides derived from antimycins) and it would have partly got the upper hand on the initial PPTase (SCO6673-like?) initially responsible for these activations. Indeed, we have demonstrated that SCO6673-like PPTase is also essential for the production of antimycins, suggesting that the two PPTases participate in the activation of the different carrier proteins encoded within the antimycin cluster. One could be responsible for the activation of PCP while the other could be responsible for the activation of ACP or they could act as heteromeric complex on both carrier proteins. Alternatively, *samL0372* (and *samL0373*) could have been lost in the clusters of *S. albus* J1074 and *Streptomyces* S4. Indeed, analysis of the region encompassing the gene encoding the most similar protein of SAML0372, SHJG_1263 from *Streptomyces hygroscopicus jinggansis* 5008 (Fig. 2), revealed that this gene is located within a cluster likely responsible for the production of antimycins (Accession number NC_017765, Fig. S11). A similar situation is observed in the proposed antimycin cluster of *Streptomyces blastmyceticus* (Accession number AB727666, Fig. 2; Fig. S11). Nevetherless, no orthologue of *samL0373* is present in these clusters suggesting that several rearrangements might have occurred at this locus. It will be interesting to test if SAML0373 is also involved in the antimycin production although it is hard to speculate about the role of this protein which contains an MT0933 antitox-like domain (Pfam14013).

## Supporting Information

Figure S1 **Sequence alignment of the SCO4744-like protein (ACPS) of** ***S. ambofaciens*** **ATCC23877 with** ***Streptomyces*** **ACPS-type proteins.** The aa residues conserved in at least 80% of the proteins are shaded in black.
(PDF)

Figure S2 **Sequence alignment of SAMT0172 (AlpN) of** ***S. ambofaciens*** **ATCC23877 with the most similar Sfp-type PPTases from actinomycetes.** The aa residues conserved in at least 8/9 proteins are black shaded. SAMT0172 belongs to the W/KEA subfamily and the motifs characteristic of this subfamily [9] are red boxed.
(PDF)

Figure S3 **Analysis of the spiramycin production in the ΔΔ** ***alpN*** **mutant strain.** Spiramycin production was analyzed by HPLC directly from a supernatant sample collected from a culture of the ΔΔ*alpN* mutant in MP5 liquid medium. A linear gradient from 5% to 75% acetonitrile was applied in the presence of 0.1% of trifluoroacetic acid for 70 min with a flow rate of 0.25 ml/min at a temperature of 30°C. Absorption was monitored at 232 nm. The insert shows the characteristic UV spectrum (from 200 to 350 nm) of spiramycin. The peak corresponding to the UV spectrum is labeled with an asterisk. Several peaks correspond to spiramycin (spiramycin is a mixture of three forms).
(PDF)

Figure S4 **Analysis of the stambomycin production in the ΔΔ** ***alpN*** **mutant strain by LC-MS.** Stambomycin production was analyzed from methanolic mycelium extracts of a culture of the ΔΔ*alpN*/OE484 (in purple) and ΔΔ*alpN*/pIB139 (in green) strains grown in MP5 liquid medium. On the bottom of the figure, the MS chromatogram shows the characteristic mass of the doubly charged peaks (673 and 680) and of the mono charged peaks (1363 and 1377).
(PDF)

Figure S5 **Analysis of blastmycinone and butenolide production in** ***S. ambofaciens*** **ATCC23877 and in the ΔΔ** ***alpN*** **and Δ** ***samL0372*** **mutant strains by GC-MS.** Total ion chromatograms of head space extracts from *S. ambofaciens* ATCC23877 (A), from the ΔΔ*alpN* mutant (B) and from the Δ*samL0372* mutant (C) grown on SFM agar plates. The structures of the butenolides (1–11) and blastmycinones (A–K) detected in the extracts are shown.
(PDF)

Figure S6 **Analysis of the production of antibiotics in the** ***S. ambofaciens*** **strain deleted for** ***samL0372*** **.** (A) Orange pigment and (B) kinamycin production were assessed on R2 plates in the WT and Δ*samL0372* strains. For the pigment, the photo was taken from below the plate. Inhibition of *B. subtilis* growth was visualized by the dark halo surrounding the agar plug. (C) HPLC analysis of the production of spiramycin and congocidine. Production was analyzed directly from a supernatant sample (100 µl) collected from a culture of the WT and Δ*samL0372*::apra-oriT strains in MP5. A linear gradient from 5% to 75% acetonitrile was applied in the presence of 0.1% of trifluoroacetic acid for 70 min with a flow rate of 0.25 ml/min at a temperature of 30°C. Commercial spiramycin and congocidine (100 µl at 0.1 mg/ml) were used as control. Absorption was monitored at 232 nm (spiramycin and congocidine) and 297 nm (congocidine). The inserts show UV spectra (from 200 to 350 nm) of the spiramycin and the congocidine. The peaks corresponding to the UV spectra are labeled with asterisks or black dots. Several peaks correspond to spiramycin (spiramycin is a mixture of three forms).
(PDF)

Figure S7 **Sequence alignment of SCO6673-like of** ***S. ambofaciens*** **ATCC23877 with Sfp-type PPTases from Streptomycetes.** The prototype Sfp proteins, Sfp from *B. subtilis* and *EntD* from *E. coli*, are included in the alignment. The aa residues conserved in at least 10/12 proteins are shaded in black. SCO6673-like belongs to the F/KES subfamily. The motifs characteristic of this subfamily are red boxed and the asterisks indicate residues implicated in stability or activity roles [9].
(RTF)

Figure S8 **Scanning electron micrograph of the surfaces of the** ***S. ambofaciens*** **ATCC23877 wild-type and Δ** ***sco6673-like*** **colonies.** All strains were grown at 30°C for 6 days on SFM agar plates. To prepare specimens, agar plugs were fixed with 2% osmium tetroxide for 40 h and then dehydrated by air-drying. Each specimen was sputter-coated on platinum/gold and examined with a CAMBRIDGE Stereoscan S240 scanning electron microscope. Bars: 10 µm.
(PDF)

Figure S9 **Effect of the deletion of** ***sco6673-like*** **on the congocidine production.** Congocidine bioassay was carried out on HT agar plates. After 5 days of growth at 30°C of the *Streptomyces sco6673-like* mutants, the plate was overlaid with soft nutrient agar containing *E. coli* as indicator strain. The production of congocidine was visualized by the inhibition of the indicator microorganism growth. Inhibition is only detectable for the complemented mutant strain (Δ*sco6673-like*/pIB*sco6673-like*).
(PDF)

Figure S10 **MS spectra of the stambomycins and MS and MS2 spectra of coelichelin from** ***S. ambofaciens*** **ATCC23877.** (A) MS spectra of stambomycins C/D (top) and stambomycins A/B (bottom) corresponding to the peaks of interest on the ion chromatogram for the ATCC/OE484 strain (see Fig. 6). The [M+2H-H_2_O]^2+^, [M+2H]^2+^ and [M+H]^+^ forms of stambomycins C/D (m/z 673, 682 and 1363, respectively) and stambomycins A/B (m/z 680, 689 and 1377, respectively) are indicated. (B) MS and MS2 spectra of [M+H]^+^ ion of coelichelin (desferri- form) from the WT extract (see Fig. 6). The spectra are consistent with published MS and MS2 spectra [53].
(PDF)

Figure S11 **Alignment of antimycin biosynthetic gene clusters.** The first characterized antimycin biosynthetic gene cluster, the one of the symbiont *Streptomyces* S4 [43] is used as reference for the annotation. The PPTase encoding genes, which are present in the cluster of *S. ambofaciens* ATCC23877 (AM238663), *S. hygroscopicus subsp. jinggangensis* 5008 (NC_017765) and *S. blastmyceticus* (AB727666) but absent in the cluster of *Streptomyces* S4, are labeled with a white asterisk within the ORF. The black asterisk indicates the *samL0373* gene which appears to be specific of *S. ambofaciens*. The potential targets of the SAML0372 PPTase (but also of SCO6673-like) are the ACP and PCP domains encoded by the PKS and NRPS genes, respectively and the product (PCP) of the *antG* orthologue which is conserved within all the antimycin biosynthetic gene clusters. The comparison of the clusters was done by antiSMASH [54] using the cluster of *S. hygroscopicus subsp. jinggangensis* 5008 as a query.
(PDF)

Table S1 **Oligonucleotide primers used in this work.**
(PDF)

Text S1 **Mass spectrometric methods.**
(PDF)
