# Supplementary material for: A Single Sfp-Type Phosphopantetheinyl Transferase Plays a Major Role in the Biosynthesis of PKS and NRPS Derived Metabolites in Streptomyces ambofaciens ATCC23877
Source: PLoS One. 2014 Jan 31;9(1):e87607. doi: 10.1371/journal.pone.0087607 (PMC3909215; doi:10.1371/journal.pone.0087607)
Supplement: Figure S1 — Sequence alignment of the SCO4744-like protein (ACPS) of S. ambofaciens ATCC23877 with Streptomyces ACPS-type proteins. The aa residues conserved in at least 80% of the proteins are shaded in black. (PDF) [file pone.0087607.s001.pdf]

```
                                       10        20        30        40        50        60        70        80        90       100
                               ....|....|....|....|....|....|....|....|....|....|....|....|....|....|....|....|....|....|....|....|
ACPS                           MSIIGVGIDVAEVERFGVSLERTPAMARRLFLESELLLPSGERRGIASLAARFAAKEALAKALGAPAGLLWTDAEVYVEDSGQPRLRVLGTVAARAAELG
S. turgidiscabies Car8         MSIIGVGIDVAEIERFRASLERTPALAQRLFLESELRLPSGERRGVASLAARFAAKEAVAKALGAPAGLLWTDAEVCVEDSGRPWLRVTGTVAARAAELG
Streptomyces sp. e14           MSIIGVGIDVAEIDRFAASLERTPGLVARLFVERELLLPSGERRGMASLAARFAAKEAVAKALGAPAGLLWTDAEVYVEDSGRPRLRVKGSVAARAAELG
S. coelicoflavus ZG0656        MSIIGVGIDVAEVERFGASLERTPALAGRLFVESELLLPSGGRRGVASLAARFAAKEALAKALGAPAGLLWTDAEVYVEDSGQPRLRVTGTVAARAAELG
S. chattanoogensis             -MIIGVGIDVAEIDRFGAALERTPELAQRLFIEAELMLPSGERRGVASLAARFAAKEAVAKALGAPGGLHWTDAEVYVEDSGRPRLRVRGTVAACAAELG
S. somaliensis DSM 40738       -MIIGVGIDVAEIDRFAEALERTPQLAERLFLERELCLPSGERRGVASLAARFAAKEAVAKALGAPAGLHWTDAEVYVEESGQPRLRVRGTVAARAAELG
S. griseoflavus Tu4000         MSIIGVGIDVAEVERFAESLERTPALAQRLFLESELLLPSGERRGIASLAARFAAKEALAKALGAPAGLYWTDAEVWCEDSGQPRLRVKGTVAARAAELG
S. coelicolor A3(2)            MSIIGVGIDVAEVERFGAALERTPALAGRLFLESELLLPGGERRGVASLAARFAAKEALAKALGAPAGLLWTDAEVWVEAGGRPRLRVTGTVAARAAELG
S. ghanaensis ATCC 14672       MSIIGVGIDVAEIERFAASLERTPGLARRLFVEGELLLPSGERRGVASLAARFAAKEALAKALGAPSGLYWTDAEVWCEDSGQPRLRVKGTVAARAAELG
S. albus J1074                 -MIIGVGIDVAEIERFAASVQRTPALLDRLFVPAELVTPSGDRRGPASLAARFAAKEAVAKALGAPAGLLWTDCEVYAEDTGRPRLRVRGTVAARAAELG

                                      110       120
                               ....|....|....|....|...
ACPS                           VASWHVSLSHDAGIASAVVIAEG
S. turgidiscabies Car8         VRSWHVSLSHDAGVASAVVIAEA
Streptomyces sp. e14           VRAWHISLSHDAGVASAVVIAEG
S. coelicoflavus ZG0656        VASWHVSLSHDAGIASAVVIAEG
S. chattanoogensis             VRSWHVSLSHDAGVASAVVIAEG
S. somaliensis DSM 40738       VRHWHVSLSHDAGVASAVVIAEG
S. griseoflavus Tu4000         VRSWHVSLSHDAGIASAVVIAEG
S. coelicolor A3(2)            VASWHVSLSHDAGIASAVVIAEG
S. ghanaensis ATCC 14672       VRSWHVSLSHDAGVASAVVIAEG
S. albus J1074                 VTGWHVSLSHDAGVASAVVIAEG
```

Figure S1. Sequence alignment of the SCO4744-like protein (ACPS) of *S. ambofaciens* ATCC23877 with *Streptomyces* ACPS-type proteins.

The aa residues conserved in at least 80% of the proteins are shaded in black.
