# Supplementary material for: A Single Sfp-Type Phosphopantetheinyl Transferase Plays a Major Role in the Biosynthesis of PKS and NRPS Derived Metabolites in Streptomyces ambofaciens ATCC23877
Source: PLoS One. 2014 Jan 31;9(1):e87607. doi: 10.1371/journal.pone.0087607 (PMC3909215; doi:10.1371/journal.pone.0087607)
Supplement: Figure S2 — Sequence alignment of SAMT0172 (AlpN) of S. ambofaciens ATCC23877 with the most similar Sfp-type PPTases from actinomycetes. The aa residues conserved in at least 8/9 proteins are black shaded. SAMT0172 belongs to the W/KEA subfamily and the motifs characteristic of this subfamily [9] are red boxed. (PDF) [file pone.0087607.s002.pdf]

```
                                        10        20        30        40        50        60        70        80        90       100
                                ....|....|....|....|....|....|....|....|....|....|....|....|....|....|....|....|....|....|....|....|
SAMT0172 [AlpN]                 ------------------------------------------------MDRVTCAVPLHVP---RPSGPWPGVRENLSRLGNALVCTTWSEWLP----SV
S. pristinaespiralis ATCC25486  ------------------------------------------------MNEQRISTPIHVP---RPAGPWQGVHENLTRTGNAVVHTTWGEWLP----AA
S. rochei                       ------------------------------------------MTTPVHTGAGRIGAPFHVS---GPDAPWHRVRAAMDRHGTAVVHTTWGEWLA----AV
Streptomyces sp. SM8            -------------------------------------------MSHRSDVHHPSEMPLHMPG-SAPGRPWEAVRARLEREGHVVVTATWGEWLS----AA
S. coelicoflavus ZG0656        MSPDLDRNDSAPPVRGGSAGVTDDIDARDAGGVLGTLRRLGDLGVVVTAHGRAVAPPVHVP---RPDSSWLRLREDLLDRGVTVAYATWTEWFP----AV
S. vitaminophilus               ---------------------------------MASTRAVIHPFGNPPAQQPAVAEPVRVRG---TAGPWDRVRHDLASLGWAVLHARIDDWLP----HP
S. ghanaensis ATCC 14672        -------------------------------------------------MSSTISEPLQVTP----LGPWHTVRRELAEQGSVVVFARLDDWLTDWQPDT
Kitasatospora setae KM-6054     ----------------------------------------MIPLRD---ARAAIGEPIAVAPG--PASGWERVREALAVRGSAVLVARLGEWLPAAGADP
Streptomyces sp. JP95           --------------------------------------------------MPRLAPPVHVG---GPDGPWNQLHRQWEDSGGVVVLATWGEWLP----AV

                                       110       120       130       140       150       160       170       180       190       200
                                ....|....|....|....|....|....|....|....|....|....|....|....|....|....|....|....|....|....|....|....|
SAMT0172 [AlpN]                 LTTRRLPQLLGADWPRYRRTPDAAVRYRFAASRLLIKNTAAAALAVPPEDLDLAYRLGGRPYLRGFDQIELSLSHTGDLIAVGLSRTGRIGVDVEPAERP
S. pristinaespiralis ATCC25486  LTAPRLRRLLGRDWTRYRRIPDATVRFRFVAARLLMKHTAAAALRVDPETLDLAYRLGGRPYLRGFDQIDLSLTHTGDLMAVGLSRHGRIGVDAEPADRR
S. rochei                       VAEPALRPLLGRDWQRYRRTADPTVRYRFVASRLVTKYTAAAALHTEPVELDLAYRTGGRPYLRGLDQIDVSLTHTDDLIAVGISRVGRIGVDAEPAGRR
Streptomyces sp. SM8            LTAPALRPLLGRDWQRYRRTENAVARYRFVTSRMLVKHTAAAVLDSDPAALELAYRIGGRPFLRGFDQLDVSLSHTGDLLVVAVSRTGRIGVDAEPVDRE
S. coelicoflavus ZG0656        LTTPRLRPLLGQDWNRYTATRDPAVRCRFAASRLLIKYTAAAALGIGPEDIDIAYQLGGRPYLRGLDQIEVALTHTGELMAVGISRVGRIGLDAECADRR
S. vitaminophilus               AHTPSLKRLLGRDWERYLRMRNPRVRARFAATRSLLKHAAGVAVQAPPHTVELAYTAFGRPYLRGLDQVDVSLSHTGDFLLVGLTTRGRIGVDAEKIDRP
S. ghanaensis ATCC 14672        GADSALPALLGRDWERFQKMTNEDVRARFLASRLLLKYAAGAVLEARPVDIDLAYKPGGRPYLRGCGQVDISLSHTADLLVVGITRQGWIGVDAELTGRQ
Kitasatospora setae KM-6054     APETGYRAVLGRDWNRYRAITHSQVRERFLASRMLLKRAAGEALQAPPESVDLAYKLGGRPYLRGCDQVDISLSHTAELLVVGLTRRGWIGVDTELDDRV
Streptomyces sp. JP95           VTDPRLRGLLGDDWPRYRRTADATVRHRFAAARFVLKHAAAAALETDPAELDLAYRPGGRPYLRGVRGVDVSLAHTGELVVVGLSRTGRIGVDAEPLTRS
                                                                                                          1                2

                                       210       220       230       240       250       260       270       280       290       300
                                ....|....|....|....|....|....|....|....|....|....|....|....|....|....|....|....|....|....|....|....|
SAMT0172 [AlpN]                 VRLELLQTQVFTPAEARELASLP----ENERPGRALRIWTLKEAYSKALGQGLRLGFKEFGF--IDGRL---------STPDGSAVADGEWGFATYPVMD
S. pristinaespiralis ATCC25486  MRFDLLQAQMCTPTEAAELAPLP----EREQTAQTLRIWTLKEAYTKALGQGMRLGFTEFGFGLRSGRL---------LAPDGSPATRGEWAFATHPALG
S. rochei                       MSYELLGRTVLTPAERAALEPLA----DAERAAAMLRIWTLKEAYTKALGQGLRLGFDEFG--FDADGS-------GLAAPDGRPVRRDAWTFATYRVLG
Streptomyces sp. SM8            IPFGVLGRRMCTPAEAAPLAGLT----PERQARVLLRIWTLKEAYTKALGQGMRLGFTEFGFG-DDGAP---------ATADGSPAPGGDWTFSTHPVLD
S. coelicoflavus ZG0656        FDLDLMRGRMCTPAEAAELSALA----RDEQAACVLRIWTLKEAYTKALGQGMRLAFTEFGFGLGEGGL---------RTPGGAAAAGEEWAFATHPVLG
S. vitaminophilus               LLRNGTERHICTPYELDMLERVP----EARRNRSAVRIWTLKEAYSKAIGQGLRFRFSEFGFGEDGQPVR-------VQRPDGTPGTGSEWTFATF-TPD
S. ghanaensis ATCC 14672        LSGSAVERQICTPHETELLARTG----EQQRNTALVRIWTLKEAYSKAIGQGLRFRFTEFGFAPGARRVQ-------VLRPDGRPGSGDEWSFCTW-SVA
Kitasatospora setae KM-6054     LLGTGAEKQICTPYEAGQLAAMA----EHRRNPALVRIWTLKEAYSKAIGQGLRFRFTEFGFTSEEAPVT-------VLRPDGSPGTGEEWSFGTW-AVD
Streptomyces sp. JP95           LSVDVLLARACTPDERAAVKTLGGIGGAAGVTAALLRIWTLKEAYTKALGCGLRLPFARFGVPWAVEGEAAAYGDEGLLSPGGGPAGPRAWTFGAHRALG
                                                                           3

                                       310       320       330       340       350
                                ....|....|....|....|....|....|....|....|....|....|....|...
SAMT0172 [AlpN]                 -RYLLSVACHDAGLSTAGDTSAGTMLDQGFLAAMTDTAPR------------------
S. pristinaespiralis ATCC25486  GRYLLSVACHDAGLDPADDTSARTMLDPGFLAAMAHHQH-------------------
S. rochei                       -RYVLSVARHDAGLDSSPDTAAHTMLDEGFMDAVGELLP-------------------
Streptomyces sp. SM8            GRYLVSTACQDTGLDPARDLTARTMLDPQFTGAVRTARHLEPGGPPRRAL--------
S. coelicoflavus ZG0656        -RYLVSTACHDAGLDPDGDTSVGTMLDRRLLTAMTPPGEQGPGGDQGPPGERGPVADF
S. vitaminophilus               QQYCASVAVHDTGFGAPADTAAATMLDSGLVDAVNELVARARSEQRAAA---------
S. ghanaensis ATCC 14672        DRYTVSAALVDLGFDEAVDVAARTMLDEGMADAL--LTQPQRTE--------------
Kitasatospora setae KM-6054     G-YTVSVALFDAGFGDAADIAALTMLDDGLVDAL--IGGPGRGPDSPT----------
Streptomyces sp. JP95           -RYLVGVARHDG----APAAPLRPSSDR----------P-------------------
```

Figure S2. Sequence alignment of SAMT0172 (AlpN) of *S. ambofaciens* ATCC23877 with the most similar Sfp-type PPTases from actinomycetes.

The aa residues conserved in at least 8/9 proteins are black shaded. SAMT0172 belongs to the W/KEA subfamily and the motifs characteristic of this subfamily [9] are red boxed.
