# Supplementary material for: A Single Sfp-Type Phosphopantetheinyl Transferase Plays a Major Role in the Biosynthesis of PKS and NRPS Derived Metabolites in Streptomyces ambofaciens ATCC23877
Source: PLoS One. 2014 Jan 31;9(1):e87607. doi: 10.1371/journal.pone.0087607 (PMC3909215; doi:10.1371/journal.pone.0087607)
Supplement: Figure S3 — Analysis of the spiramycin production in the ΔΔ alpN mutant strain. Spiramycin production was analyzed by HPLC directly from a supernatant sample collected from a culture of the ΔΔalpN mutant in MP5 liquid medium. A linear gradient from 5% to 75% acetonitrile was applied in the presence of 0.1% of trifluoroacetic acid for 70 min with a flow rate of 0.25 ml/min at a temperature of 30°C. Absorption was monitored at 232 nm. The insert shows the characteristic UV spectrum (from 200 to 350 nm) of spiramycin. The peak corresponding to the UV spectrum is labeled with an asterisk. Several peaks correspond to spiramycin (spiramycin is a mixture of three forms). (PDF) [file pone.0087607.s003.pdf]

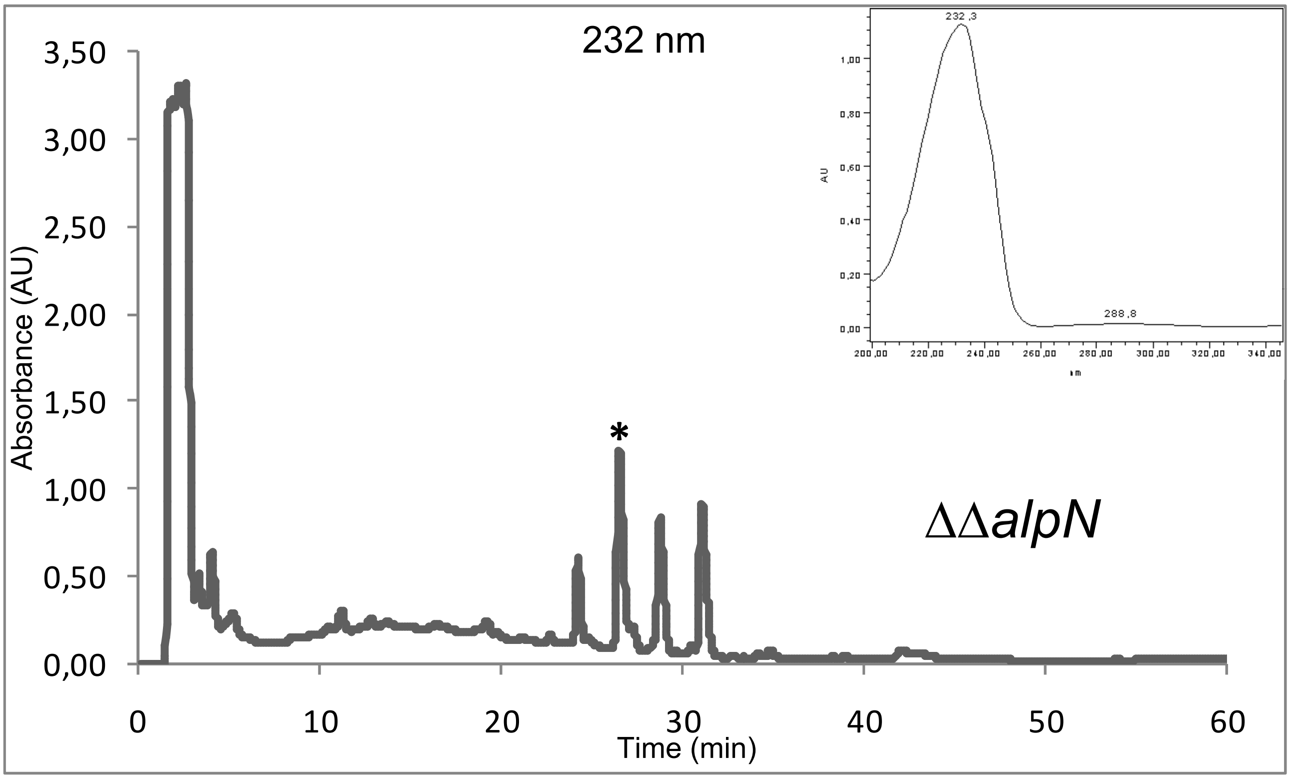

Figure S3. Analysis of the spiramycin production in the ΔΔ*alpN* mutant strain.

Spiramycin production was analyzed by HPLC directly from a supernatant sample collected from a culture of the ΔΔ*alpN* mutant in MP5 liquid medium. A linear gradient from 5 % to 75 % acetonitrile was applied in the presence of 0.1 % of trifluoroacetic acid for 70 min with a flow rate of 0.25 ml/min at a temperature of 30°C. Absorption was monitored at 232 nm. The insert shows the characteristic UV spectrum (from 200 to 350 nm) of spiramycin. The peak corresponding to the UV spectrum is labeled with an asterisk. Several peaks correspond to spiramycin (spiramycin is a mixture of three forms).
