# Supplementary material for: A Single Sfp-Type Phosphopantetheinyl Transferase Plays a Major Role in the Biosynthesis of PKS and NRPS Derived Metabolites in Streptomyces ambofaciens ATCC23877
Source: PLoS One. 2014 Jan 31;9(1):e87607. doi: 10.1371/journal.pone.0087607 (PMC3909215; doi:10.1371/journal.pone.0087607)
Supplement: Figure S4 — Analysis of the stambomycin production in the ΔΔ alpN mutant strain by LC-MS. Stambomycin production was analyzed from methanolic mycelium extracts of a culture of the ΔΔalpN/OE484 (in purple) and ΔΔalpN/pIB139 (in green) strains grown in MP5 liquid medium. On the bottom of the figure, the MS chromatogram shows the characteristic mass of the doubly charged peaks (673 and 680) and of the mono charged peaks (1363 and 1377). (PDF) [file pone.0087607.s004.pdf]

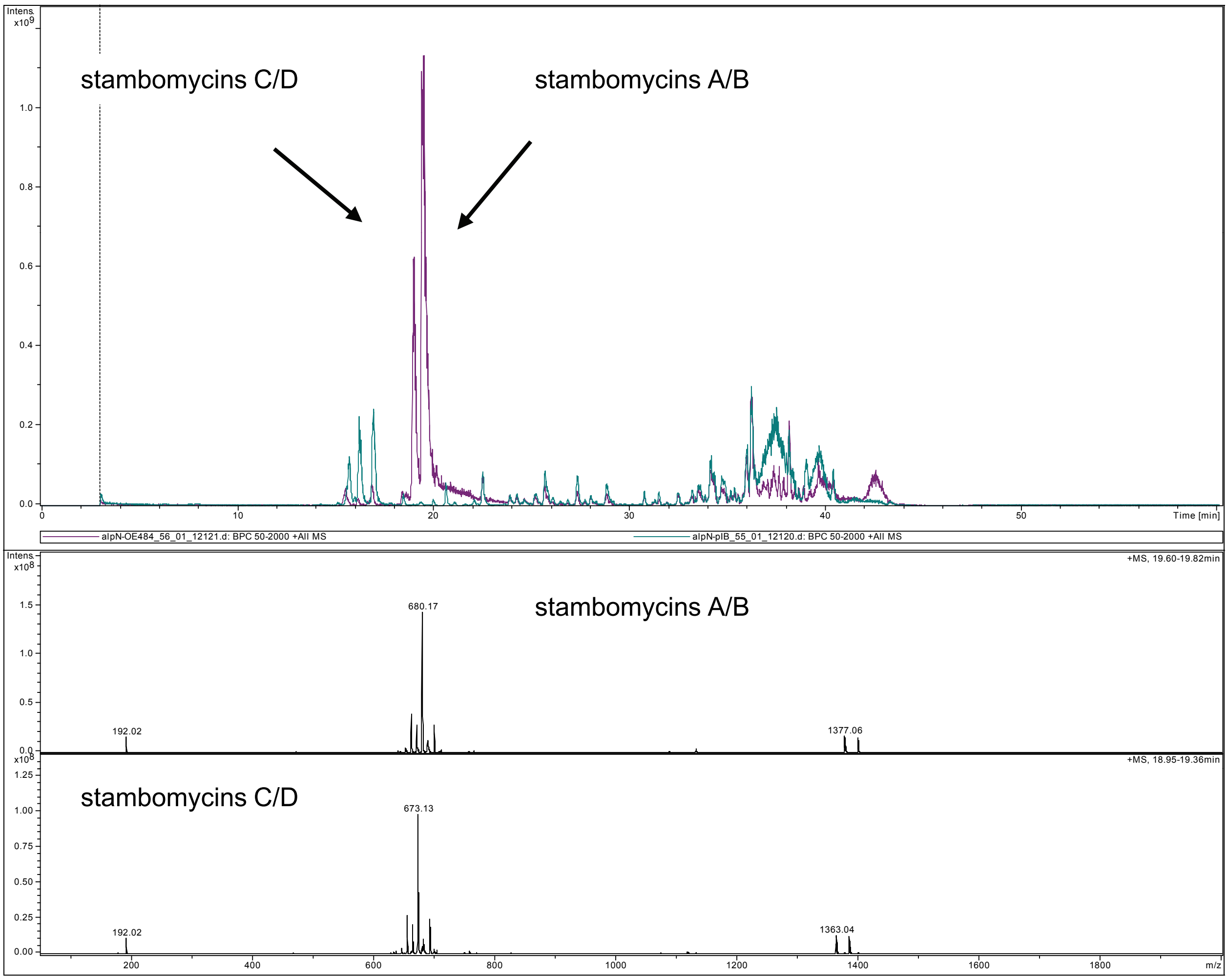

Figure S4. Analysis of the stambomycin production in the ΔΔ*alpN* mutant strain by LC-MS. Stambomycin production was analyzed from methanolic mycelium extracts of a culture of the ΔΔ*alpN*/OE484 (in purple) and ΔΔ*alpN*/pIB139 (in green) strains grown in MP5 liquid medium. On the botton of the figure, the MS chromatogram shows the characteristic mass of the doubly charged peaks (673 and 680) and of the mono charged peaks (1363 and 1377).
