# Supplementary material for: A Single Sfp-Type Phosphopantetheinyl Transferase Plays a Major Role in the Biosynthesis of PKS and NRPS Derived Metabolites in Streptomyces ambofaciens ATCC23877
Source: PLoS One. 2014 Jan 31;9(1):e87607. doi: 10.1371/journal.pone.0087607 (PMC3909215; doi:10.1371/journal.pone.0087607)
Supplement: Figure S5 — Analysis of blastmycinone and butenolide production in S. ambofaciens ATCC23877 and in the ΔΔ alpN and Δ samL0372 mutant strains by GC-MS. Total ion chromatograms of head space extracts from S. ambofaciens ATCC23877 (A), from the ΔΔalpN mutant (B) and from the ΔsamL0372 mutant (C) grown on SFM agar plates. The structures of the butenolides (1–11) and blastmycinones (A–K) detected in the extracts are shown. (PDF) [file pone.0087607.s005.pdf]

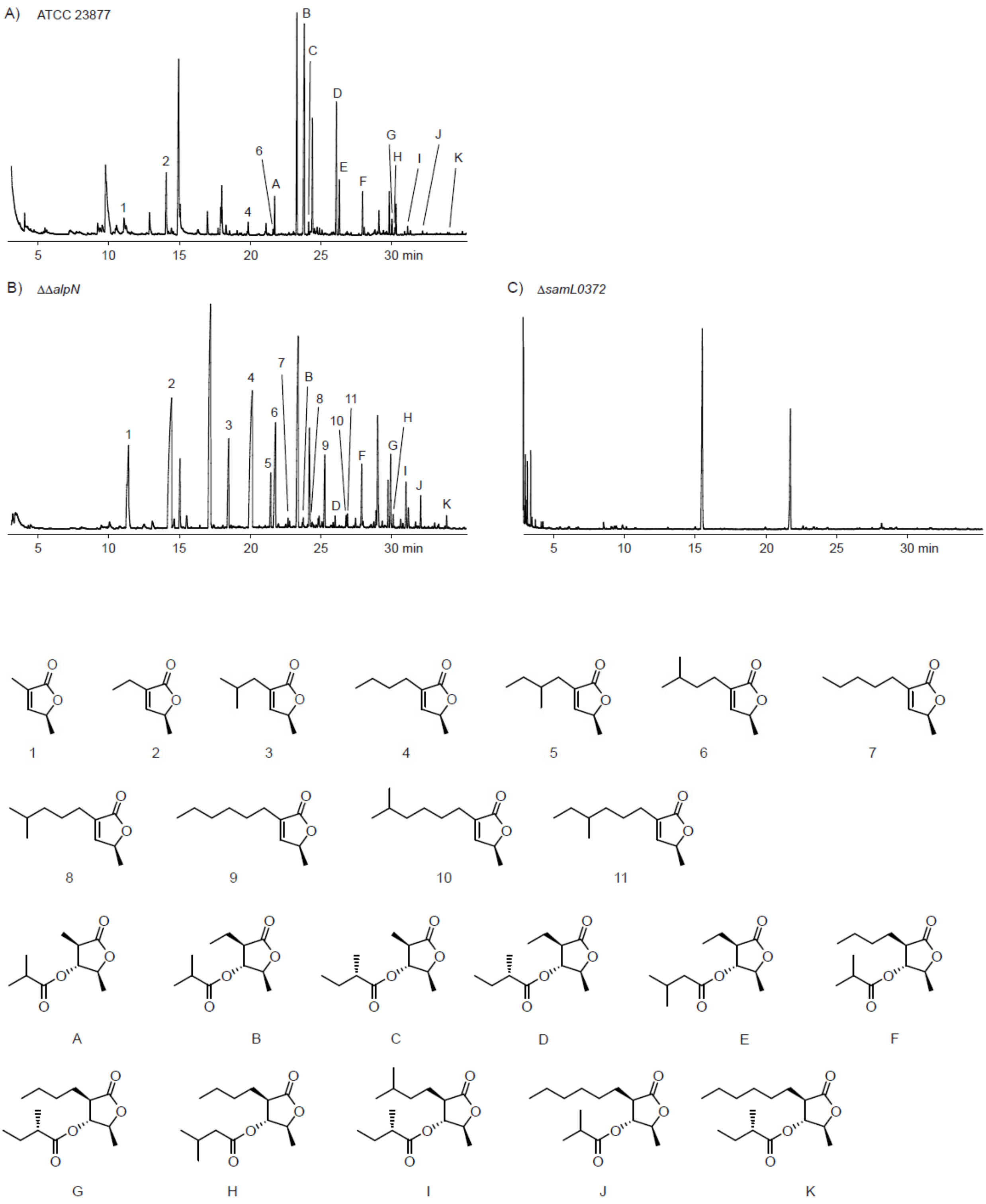

Figure S5. Analysis of blastmycinone and butenolide production in *S. ambofaciens* ATCC23877 and in the ΔΔ*alpN* and Δ*samL0372* mutant strains by GC-MS.

Total ion chromatograms of head space extracts from *S. ambofaciens* ATCC23877 (A), from the ΔΔ*alpN* mutant (B) and from the Δ*samL0372* mutant (C) grown on SFM agar plates. The structures of the butenolides (1-11) and blastmycinones (A-K) detected in the extracts are shown.
