# Supplementary material for: A Single Sfp-Type Phosphopantetheinyl Transferase Plays a Major Role in the Biosynthesis of PKS and NRPS Derived Metabolites in Streptomyces ambofaciens ATCC23877
Source: PLoS One. 2014 Jan 31;9(1):e87607. doi: 10.1371/journal.pone.0087607 (PMC3909215; doi:10.1371/journal.pone.0087607)
Supplement: Figure S6 — Analysis of the production of antibiotics in the S. ambofaciens strain deleted for samL0372 . (A) Orange pigment and (B) kinamycin production were assessed on R2 plates in the WT and ΔsamL0372 strains. For the pigment, the photo was taken from below the plate. Inhibition of B. subtilis growth was visualized by the dark halo surrounding the agar plug. (C) HPLC analysis of the production of spiramycin and congocidine. Production was analyzed directly from a supernatant sample (100 µl) collected from a culture of the WT and ΔsamL0372::apra-oriT strains in MP5. A linear gradient from 5% to 75% acetonitrile was applied in the presence of 0.1% of trifluoroacetic acid for 70 min with a flow rate of 0.25 ml/min at a temperature of 30°C. Commercial spiramycin and congocidine (100 µl at 0.1 mg/ml) were used as control. Absorption was monitored at 232 nm (spiramycin and congocidine) and 297 nm (congocidine). The inserts show UV spectra (from 200 to 350 nm) of the spiramycin and the congocidine. The peaks corresponding to the UV spectra are labeled with asterisks or black dots. Several peaks correspond to spiramycin (spiramycin is a mixture of three forms). (PDF) [file pone.0087607.s006.pdf]

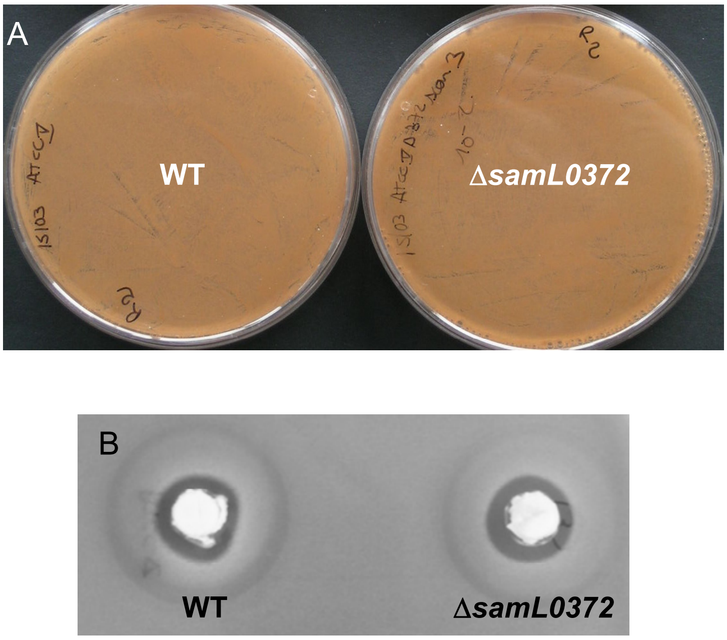
A
WT
ΔsamL0372
B
WT
ΔsamL0372

C

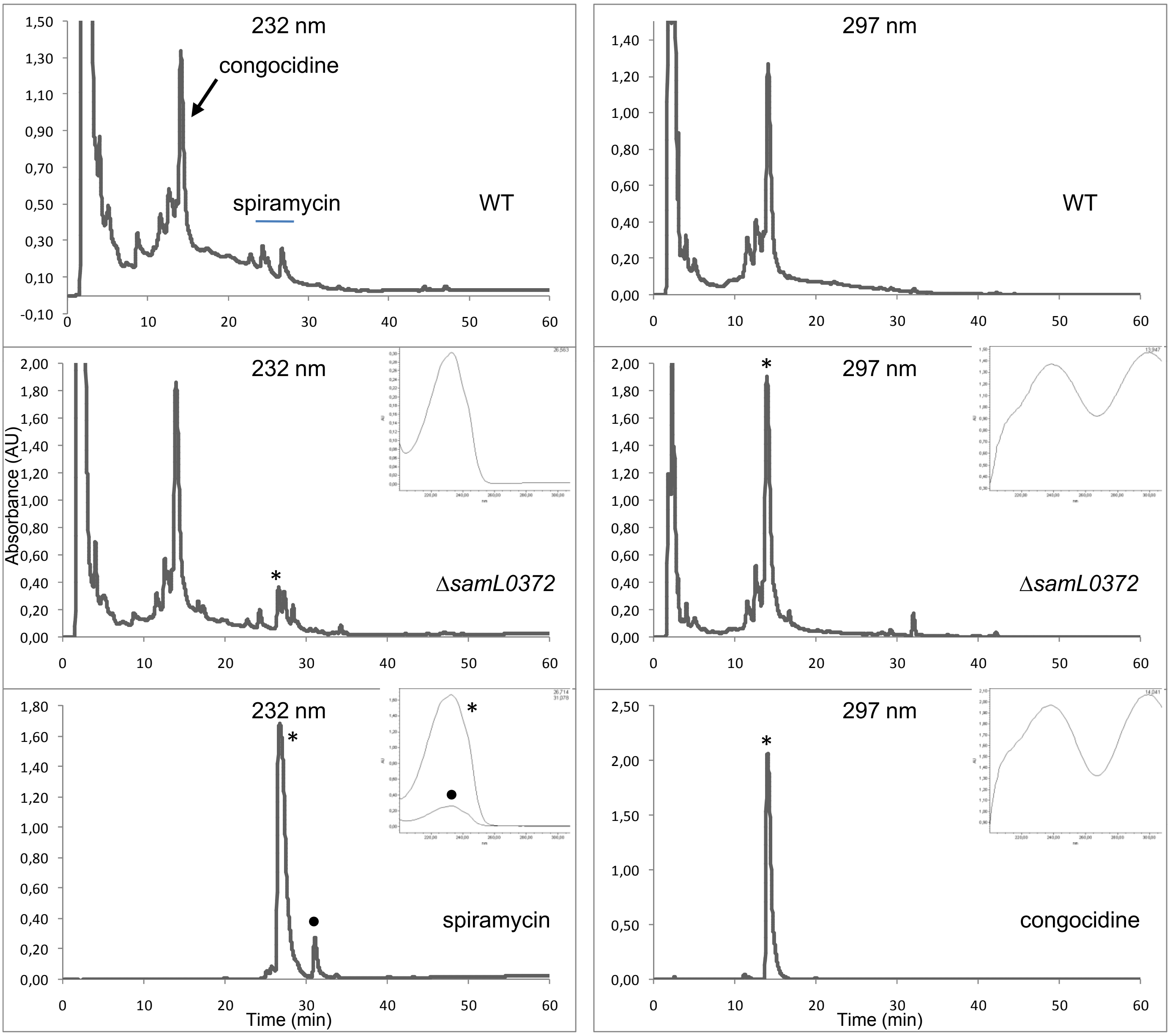

Figure S6. Analysis of the production of antibiotics in the *S. ambofaciens* strain deleted for *samL0372*.

(A) Orange pigment and (B) kinamycin production were assessed on R2 plates in the WT and Δ*samL0372* strains. For the pigment, the photo was taken from below the plate. Inhibition of *B. subtilis* growth was visualized by the dark halo surrounding the agar plug. (C) HPLC analysis of the production of spiramycin and congocidine. Production was analyzed directly from a supernatant sample (100 μl) collected from a culture of the WT and Δ*samL0372*::apra-oriT strains in MP5. A linear gradient from 5% to 75% acetonitrile was applied in the presence of 0.1% of trifluoroacetic acid for 70 min with a flow rate of 0.25 ml/min at a temperature of 30°C. Commercial spiramycin and congocidine (100 μl at 0.1 mg/ml) were used as control. Absorption was monitored at 232 nm (spiramycin and congocidine) and 297 nm (congocidine). The inserts show UV spectra (from 200 to 350 nm) of the spiramycin and the congocidine. The peaks corresponding to the UV spectra are labeled with asterisks or black dots. Several peaks correspond to spiramycin (spiramycin is a mixture of three forms).
