# Supplementary material for: A Single Sfp-Type Phosphopantetheinyl Transferase Plays a Major Role in the Biosynthesis of PKS and NRPS Derived Metabolites in Streptomyces ambofaciens ATCC23877
Source: PLoS One. 2014 Jan 31;9(1):e87607. doi: 10.1371/journal.pone.0087607 (PMC3909215; doi:10.1371/journal.pone.0087607)
Supplement: Figure S7 — Sequence alignment of SCO6673-like of S. ambofaciens ATCC23877 with Sfp-type PPTases from Streptomycetes. The prototype Sfp proteins, Sfp from B. subtilis and EntD from E. coli, are included in the alignment. The aa residues conserved in at least 10/12 proteins are shaded in black. SCO6673-like belongs to the F/KES subfamily. The motifs characteristic of this subfamily are red boxed and the asterisks indicate residues implicated in stability or activity roles [9]. (RTF) [file pone.0087607.s007.rtf]

                                     10        20        30        40        50        60        70        80        90       100                  
                            ....|....|....|....|....|....|....|....|....|....|....|....|....|....|....|....|....|....|....|....|
SCO6673-like                -----MIEDLLPATVVTVEAHGHDDAGHLPLYPEEEALVARAVDKRRREFTAVRSCARRAMEKLGVPAQPVLNGERGAPRWPSGLVGSMTHCDGYCAAAL 
S. coelicoflavus ZG0656     -----MIEELLPDTVVAVEACGHDDAGHLPLYPEEEAIVARAVAKRRREFTVVRSCARRAMEKLGVPPQPVPTGERGAPRWPAGLAGSMTHCDGYCAAAL 
S. coelicolor A3(2)         -----MIEELLPGTVVAVEAFGQDDAGHLPLYPEEEELVARAVAKRRREFTVVRSCARRAMEKLGVPAQPVLTGERGAPRWPEGIAGSMTHCDGYGAAAL 
S. lividans TK24            -----MIEELLPGTVVAVEAFGQDDAGHLPLYPEEEELVARAVAKRRREFTVVRSCARRAMEKLGVPAQPVLTGERGAPRWPEGIAGSMTHCDGYGAAAL 
S. chartreusis NRRL 12338   -----MIGELLPQTVVTVEAYG-NDGADTPLYPEEAALVTRAVAKRRREFTVVRSCARRAMEKLGVPPQPILPGERGAPRWPAGLAGSMTHCDGYCAAAL 
S. sviceus ATCC 29083       -----MIEELLPDSVVAVEVHGHDEPGNTALYPEEAALVALAVPKRRHEFAVVRACARRAMEKLGVPPQPVLSGERGAPRWPAGLTGSMTHCDGYCAAAL 
S. davawensis JCM 4913      -----MIEELLPDSVVTVEAYGEEDASPAVLYPEEAAVVARAVDKRRREFAAVRVCARRAMEKLGVPAQPLLPGDRGAPRWPDGLAGSMTHCDGYCAAAL 
S. ghanaensis ATCC 14672    -----MIEELLPRTVVVVEAYG-DDGADAPLYPEEAALLTRAVPKRRREFAAVRSCARRAMRKLGVPARPVLPGERGAPVWPDGLTGSMTHCEGYCAAAL 
S. viridochromogenes Tue57  -----MIEELLPDTVVAVETYGDEEPPNAALYPEEEALVAQAVAKRRREFAAVRSCARRAMEKLGVPPQPVLPGERGAPVWPAGLAGSMTHCDGYCAAAL 
S. avermitilis MA-4680      -----MIEELLPAAVVAVEAHGDEAAVDGALYPEEQAVIARAVEKRRREFTAVRVCARRAMEKLGVPPQPVLPGERGAPRWPAGLVGSMTHCEGYCAAVL 
B. subtilis Sfp             ---MKIYGIYMDRPLSQEENERFMSFISPEKREKCRRFYHKEDAHRTLLGDVLVRSVISRQYQLDKSDIRFSTQEYGKPCIPD--LPDAHFNISHSGRWV 
E. coli EntD                MKTTHTSLPFAGHTLHFVEFDPANFCEQDLLWLPHYAQLQHAGRKRKTEHLAGRIAAVYALREYGYKCVPAI-GELRQPVWPAEVYGSISHCG--TTALA 
											  1a					    1
                                    110       120       130       140       150       160       170       180       190       200         
                            ....|....|.*.*.....|....|....|.*..|....|....|....|....|**..|*...|....|....|....|....|....|....|....|
SCO6673-like                VRATDLASVGIDAEPDGPLPDGVLPGVSLPAEAERLRRLGRERPGVHWDRLLFSAKESVYKAWFPLTGEWLDFMEADIEISVDPVDP---RRGTLRAALL 
S. coelicoflavus ZG0656     VRLTDLASLGIDAEPDGPLPDGVLESVALPAETARLRRLDEARPGIHWERLLFSAKESVYKAWYPLTGRWLDFTEADIEIAVDPADP---RRGTLHAVLL 
S. coelicolor A3(2)         VRLTDLASLGIDAEPDGPLPDGVLESIALPAEVALLRRLGGARPGVHWDRLLFSAKESVYKAWYPLTGQWLDFTEADIEIRVDPADP---RRGTLHAALL 
S. lividans TK24            VRLTDLASLGIDAEPDGPLPDGVLESIALPAEVALLRRLGGARPGVHWDRLLFSAKESVYKAWYPLTGQWLDFAEADIEIRVDPADP---RRGTLHAALL 
S. chartreusis NRRL 12338   VRATDLASLGIDAEVHGPLPEGVLPAVSLPAEAERLRRLAAQRPDVHWDRLLFSAKESVYKAWFPLTGAWLDFAEADIEISADPGE----PRGTFRATLL 
S. sviceus ATCC 29083       VRSADLASLGIDAEPHGPLPEGVLESVSLPGERVRLRELARTYPDVHWDRLLFSAKESVYKAWFPLTGTWLDFSEADIDILVAPGRP---VSGTLRAELL 
S. davawensis JCM 4913      VRAADLASLGIDAEPDEPLPDGVLAAVSLPGEAQRIRRLTLDHPGIHWDRLLFSAKESVYKAWFPLTGQWLDFSEADIHLSAHPSGH---PGGTFTATLL 
S. ghanaensis ATCC 14672    VRAADLASLGIDAETHGPLPEGVLASVSLPGEAARIGRLSAERPAVHWDRLLFSAKESVYKAWFPLTGEWLDFHEADIDLFTDPGER---TRGGFRAALL 
S. viridochromogenes Tue57  VRAADLASLGIDAEPHQTLPEGVLPAVALPAEADRLRRLAGDHPGVHWDRLLFSAKESVYKAWFPLTGKWLDFGEADIDVFADRREQ---HSGGFRARLL 
S. avermitilis MA-4680      VRAGELASLGIDAEPHDRLPEGVLSSVALPTEERRLYDLGRSRPDVHWDRLLFSAKESVYKAWFPLTGKWLDFLEADIEIFTEPAAQGKALSGGFRAELL 
B. subtilis Sfp             ICAFDSQPIGIDIEKTKPISLEIAKRFFSKTEYSDLLAKD-KDEQTDYFYHLWSMKESFIKQEGKGLSLPLDSFSVRLHQDGQVSIELP---DSHSPCYI 
E. coli EntD                VVSRQPIGIDIEEIFSVQTARELTDNIITPAEHERLADCGLAFS--LALTLAFSAKESAFKASEIQT----DAGFLDYQIISWNKQQ------------- 
					     2							     3
                                    210       220       230  
                            ....|....|....|....|....|....|....
SCO6673-like                VPGPTVGGRRVGHFDGRWTARHGLVATAVTVPHA 
S. coelicoflavus ZG0656     VPGPTVGGRRLSHFDGRWTARDGLLATAITVPHT 
S. coelicolor A3(2)         VPGPTVDGRRLSRFDGRWSARDGLVTTAVTVPRT 
S. lividans TK24            VPGPTVDGRRLSRFDGRWSARDGLVTTAVTVPRT 
S. chartreusis NRRL 12338   VPGPRVGARRLGHFDGHWTADRGLVATAVAVPHD 
S. sviceus ATCC 29083       VPGPLVGGERRGVLEGRWTVRDGLVATSVTVPHA 
S. davawensis JCM 4913      VPGPLVRGHRVDVFEGRWTVERGLVATAVTVPYT 
S. ghanaensis ATCC 14672    VPGPRVGDRRLDHFDGRWTAEGGLVATAVAVPHG 
S. viridochromogenes Tue57  VPGPWVGDRRLDHFDGRWTVGRGLVATAVSVPHT 
S. avermitilis MA-4680      VPGPLVNGRRLDAFDGRWTVRRGLVATAVTVPHH 
B. subtilis Sfp             KTYEVDPGYKMAVCAAHPDFPEDITMVSYEELL- 
E. coli EntD                ----VIIHRENEMFAVHWQIKEKIVITLCQHD-- 

Figure S7. Sequence alignment of SCO6673-like of S. ambofaciens ATCC23877 with Sfp-type PPTases from Streptomycetes.
The prototype Sfp proteins, Sfp from B. subtilis and EntD from E. coli, are included in the alignment. The aa residues conserved in at least 10/12 proteins are shaded in black. SCO6673-like belongs to the F/KES subfamily. The motifs characteristic of this subfamily are red boxed and the asterisks indicate residues implicated in stability or activity roles [9].
