# Supplementary material for: A Single Sfp-Type Phosphopantetheinyl Transferase Plays a Major Role in the Biosynthesis of PKS and NRPS Derived Metabolites in Streptomyces ambofaciens ATCC23877
Source: PLoS One. 2014 Jan 31;9(1):e87607. doi: 10.1371/journal.pone.0087607 (PMC3909215; doi:10.1371/journal.pone.0087607)
Supplement: Figure S8 — Scanning electron micrograph of the surfaces of the S. ambofaciens ATCC23877 wild-type and Δ sco6673-like colonies. All strains were grown at 30°C for 6 days on SFM agar plates. To prepare specimens, agar plugs were fixed with 2% osmium tetroxide for 40 h and then dehydrated by air-drying. Each specimen was sputter-coated on platinum/gold and examined with a CAMBRIDGE Stereoscan S240 scanning electron microscope. Bars: 10 µm. (PDF) [file pone.0087607.s008.pdf]

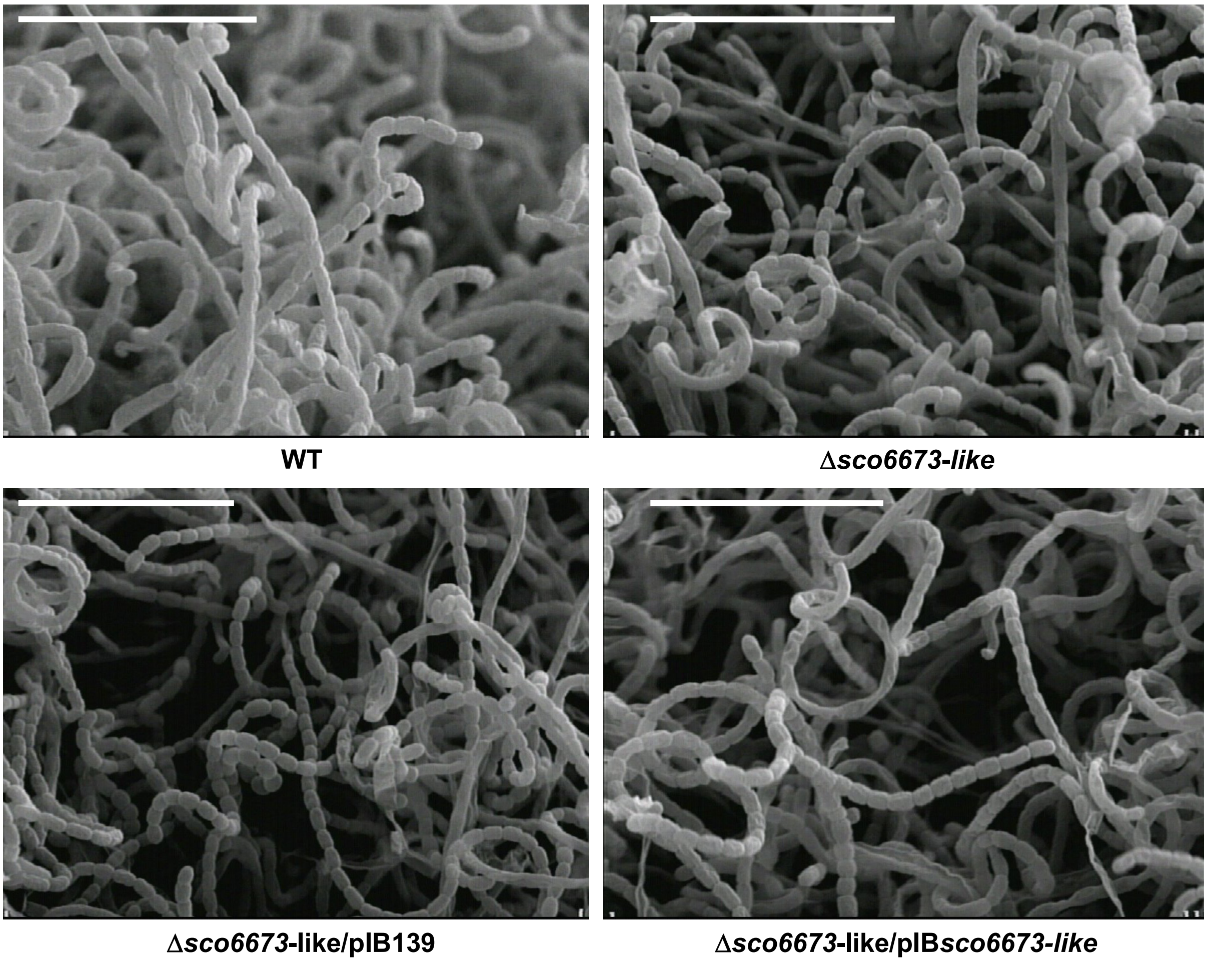

Figure S8. Scanning electron micrograph of the surfaces of the *S. ambofaciens* ATCC23877 wild-type and Δ*sco6673-like* colonies.

All strains were grown at 30°C for 6 days on SFM agar plates. To prepare specimens, agar plugs were fixed with 2% osmium tetroxide for 40h and then dehydrated by air-drying. Each specimen was sputter-coated on platinum/gold and examined with a CAMBRIDGE Stereoscan S240 scanning electron microscope. Bars: 10 μm.
