# Supplementary material for: A Single Sfp-Type Phosphopantetheinyl Transferase Plays a Major Role in the Biosynthesis of PKS and NRPS Derived Metabolites in Streptomyces ambofaciens ATCC23877
Source: PLoS One. 2014 Jan 31;9(1):e87607. doi: 10.1371/journal.pone.0087607 (PMC3909215; doi:10.1371/journal.pone.0087607)
Supplement: Figure S9 — Effect of the deletion of sco6673-like on the congocidine production. Congocidine bioassay was carried out on HT agar plates. After 5 days of growth at 30°C of the Streptomyces sco6673-like mutants, the plate was overlaid with soft nutrient agar containing E. coli as indicator strain. The production of congocidine was visualized by the inhibition of the indicator microorganism growth. Inhibition is only detectable for the complemented mutant strain (Δsco6673-like/pIBsco6673-like). (PDF) [file pone.0087607.s009.pdf]

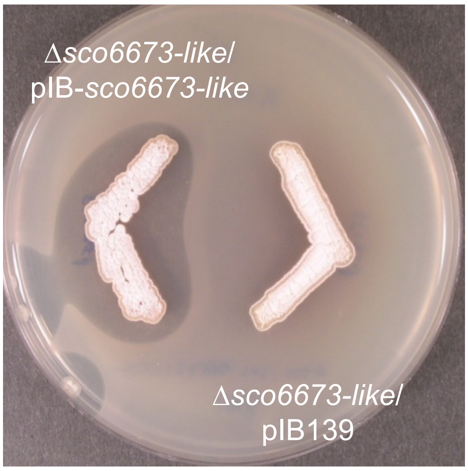

Figure S9. Effect of the deletion of *sco6673-like* on the congocidine production.
Congocidine bioassay was carried out on HT agar plates. After 5 days of growth at 30°C of the *Streptomyces sco6673-like* mutants, the plate was overlaid with soft nutrient agar containing *E. coli* as indicator strain. The production of congocidine was visualized by the inhibition of the indicator microorganism growth. Inhibition is only detectable for the complemented mutant strain (Δ*sco6673-like*/pIB*sco6673-like*).
