# Supplementary material for: A Single Sfp-Type Phosphopantetheinyl Transferase Plays a Major Role in the Biosynthesis of PKS and NRPS Derived Metabolites in Streptomyces ambofaciens ATCC23877
Source: PLoS One. 2014 Jan 31;9(1):e87607. doi: 10.1371/journal.pone.0087607 (PMC3909215; doi:10.1371/journal.pone.0087607)
Supplement: Figure S10 — MS spectra of the stambomycins and MS and MS2 spectra of coelichelin from S. ambofaciens ATCC23877. (A) MS spectra of stambomycins C/D (top) and stambomycins A/B (bottom) corresponding to the peaks of interest on the ion chromatogram for the ATCC/OE484 strain (see Fig. 6). The [M+2H-H2O]2+, [M+2H]2+ and [M+H]+ forms of stambomycins C/D (m/z 673, 682 and 1363, respectively) and stambomycins A/B (m/z 680, 689 and 1377, respectively) are indicated. (B) MS and MS2 spectra of [M+H]+ ion of coelichelin (desferri- form) from the WT extract (see Fig. 6). The spectra are consistent with published MS and MS2 spectra [53]. (PDF) [file pone.0087607.s010.pdf]

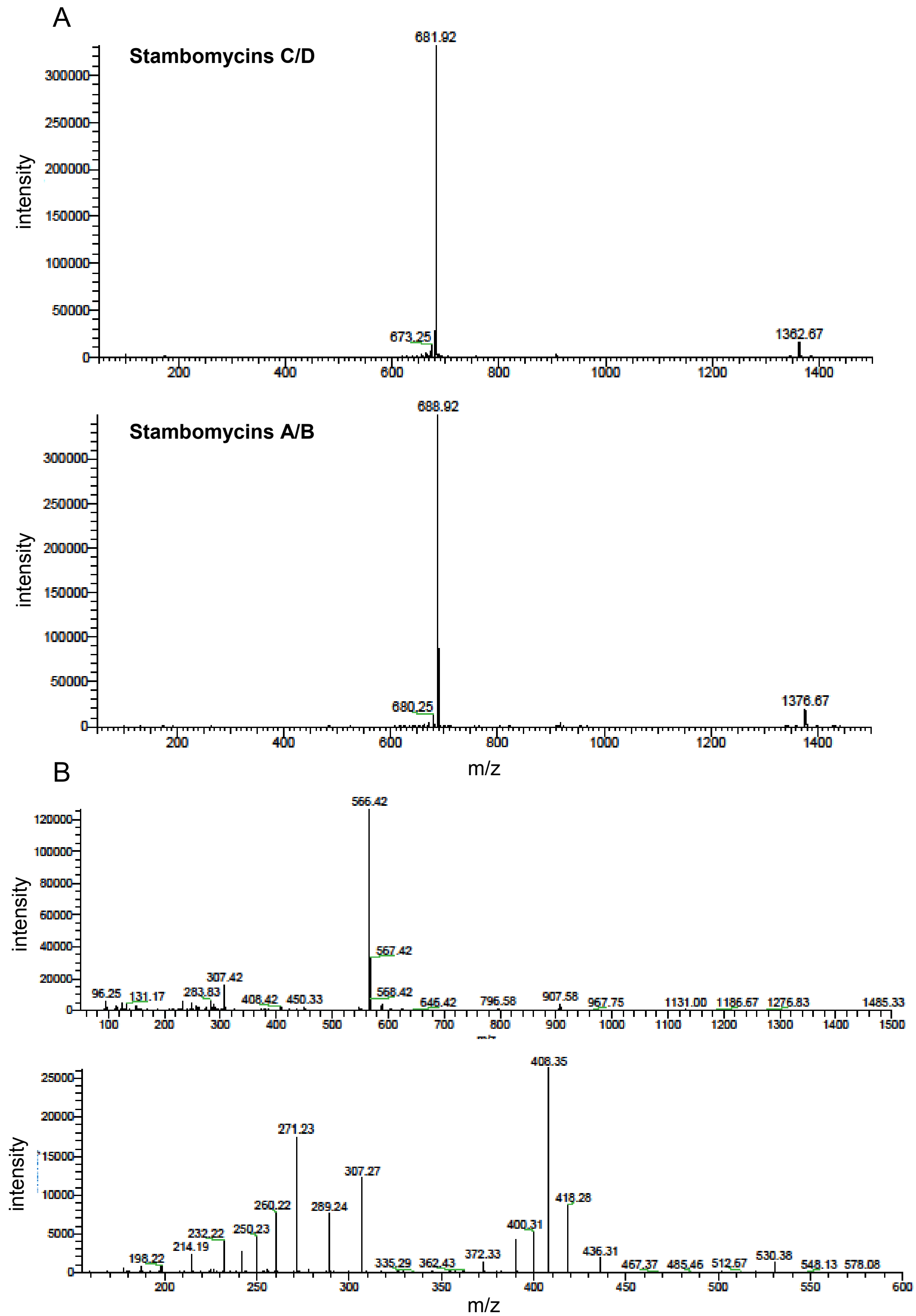

Figure S10. MS spectra of the stambomycins and MS and MS2 spectra of coelichelin from *S. ambofaciens* ATCC23877.

(A) MS spectra of stambomycins C/D (top) and stambomycins A/B (bottom) corresponding to the peaks of interest on the ion chromatogram for the ATCC/OE484 strain (see Fig. 6). The $[M+2H-H_2O]^{2+}$, $[M+2H]^{2+}$ and $[M+H]^{+}$ forms of stambomycins C/D (m/z 673, 682 and 1363, respectively) and stambomycins A/B (m/z 680, 689 and 1377, respectively) are indicated.

(B) MS and MS2 spectra of $[M+H]^{+}$ ion of coelichelin (desferri- form) from the WT extract (see Fig. 6). The spectra are consistent with published MS and MS2 spectra [52].
