# Supplementary material for: A Single Sfp-Type Phosphopantetheinyl Transferase Plays a Major Role in the Biosynthesis of PKS and NRPS Derived Metabolites in Streptomyces ambofaciens ATCC23877
Source: PLoS One. 2014 Jan 31;9(1):e87607. doi: 10.1371/journal.pone.0087607 (PMC3909215; doi:10.1371/journal.pone.0087607)
Supplement: Figure S11 — Alignment of antimycin biosynthetic gene clusters. The first characterized antimycin biosynthetic gene cluster, the one of the symbiont Streptomyces S4 [43] is used as reference for the annotation. The PPTase encoding genes, which are present in the cluster of S. ambofaciens ATCC23877 (AM238663), S. hygroscopicus subsp. jinggangensis 5008 (NC_017765) and S. blastmyceticus (AB727666) but absent in the cluster of Streptomyces S4, are labeled with a white asterisk within the ORF. The black asterisk indicates the samL0373 gene which appears to be specific of S. ambofaciens. The potential targets of the SAML0372 PPTase (but also of SCO6673-like) are the ACP and PCP domains encoded by the PKS and NRPS genes, respectively and the product (PCP) of the antG orthologue which is conserved within all the antimycin biosynthetic gene clusters. The comparison of the clusters was done by antiSMASH [54] using the cluster of S. hygroscopicus subsp. jinggangensis 5008 as a query. (PDF) [file pone.0087607.s011.pdf]

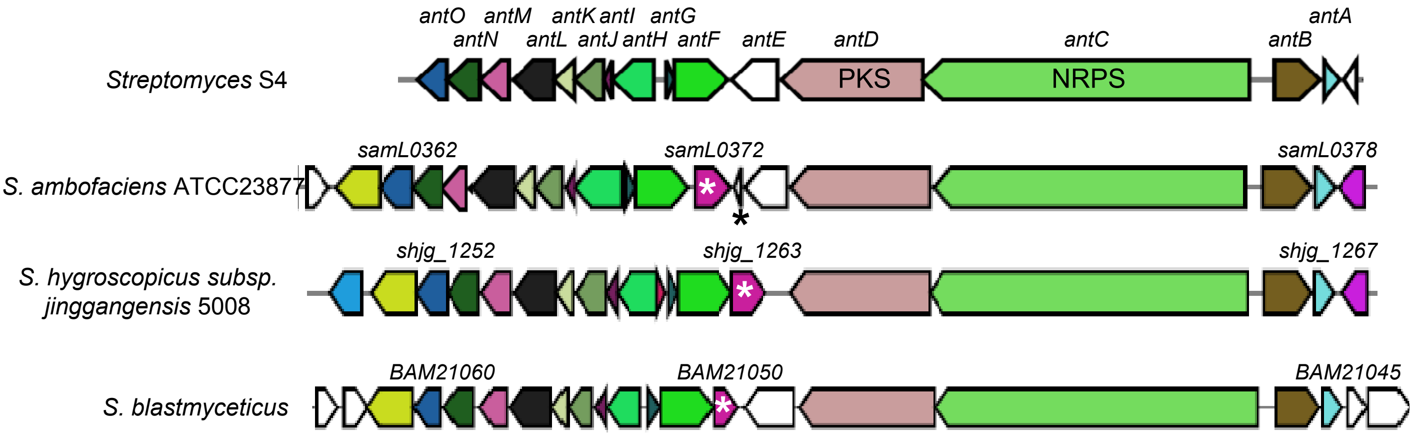

Figure S11. Alignment of antimycin biosynthetic gene clusters.
The first characterized antimycin biosynthetic gene cluster, the one of the symbiont *Streptomyces* S4 [42] is used as reference for the annotation. The PPTase encoding genes, which are present in the cluster of *S. ambofaciens* ATCC23877 (AM238663), *S. hygroscopicus subsp. jinggangensis* 5008 (NC_017765) and *S. blastmyceticus* (AB727666) but absent in the cluster of *Streptomyces* S4, are labeled with a white asterisk within the ORF. The black asterisk indicates the *samL0373* gene which appears to be specific of *S. ambofaciens*. The potential targets of the SAML0372 PPtase (but also of SCO6673-like) are the ACP and PCP domains encoded by the PKS and NRPS genes, respectively and the product (PCP) of the *antG* orthologue which is conserved within all the antimycin biosynthetic gene clusters. The comparison of the clusters was done by antiSMASH [53] using the cluster of *S. hygroscopicus subsp. jinggangensis* 5008 as a query.
