# Supplementary material for: A Single Sfp-Type Phosphopantetheinyl Transferase Plays a Major Role in the Biosynthesis of PKS and NRPS Derived Metabolites in Streptomyces ambofaciens ATCC23877
Source: PLoS One. 2014 Jan 31;9(1):e87607. doi: 10.1371/journal.pone.0087607 (PMC3909215; doi:10.1371/journal.pone.0087607)
Supplement: Table S1 — Oligonucleotide primers used in this work. (PDF) [file pone.0087607.s012.pdf]

Primers	Nucleotide sequence (5′→3′)
**Gene replacement:**	
alpN1	**GGGACCGGCGGGCGGACCACACGCAGGGAGACGACGATG**ATTCCGGGGATCCGTCGACC
alpN2	**ACGGGGCGGGGCGGTGCGGTGCGGTGCGGGGTGGTCCTA**TGTAGGCTGGAGCTGCTTC
alpN-rev*	GGCACGTCCGGGCATCAGTA
alpN-fwd*	GGCCTGCCGGAGAAACGCTT
acpS_ctg04988_F	**GCCCGACGGCGACGACGGCACCCGCCGGTCGTTAGGGTG**ATCGCGCGCGCTTCGTTCGGGACGAA
acpS_ctg04988_R	**GCTGAGCGGGGGAGGGGCCGAGGGCGGAGGGGGTGCCTA**GATCTGCCTCTTCGTCCCGAAGCAACT
SAML0372_F	**CCGGCGGGGCCTGCCGAGGCGGCCGCGGCGGCGTCGATG**ATCGCGCGCGCTTCGTTCGGGACGAA
SAML0372_R	**CCCCCCGGTGCGATGCGCCGGGGCGGCAGCCCGTGCTCA**GATCTGCCTCTTCGTCCCGAAGCAACT
amont_L0372*	GTCCGAACTGCGTCCGCT
aval_L0372*	ACGACGCCCAGACCGACAA
KO_PPT6673_fwd	**CATCCTGCCGTCGCCGCCCGGAGAGGAGACCGCCCGGTG**ATCGCGCGCGCTTCGTTCGGGACGAA
KO_PPT6673_rev	**TCAGGCGTGCGGGACCGTGACCGCCGTGGCCACCAGGCCGAT**CTGCCTCTTCGTCCCGAAGCAACT
aval_6673*	GTGCGGGACCGTGACCGC
amont_6673*	CGAGGGCGTCCGCTTCGA
**Complementation:**	
6673-comp-F	AAACATATGATCGAGGACCTGCTGCCCG
6673-comp-R	AAATCTAGATCAGGCGTGCGGGACCGTG
alpN-prom	TGACACGCACCGGAAAGGAG
alpN-fwd	GGCCTGCCGGAGAAACGCTT

Table S1. Oligonucleotide primers used in this work.
The bold nucleotides are identical to the sequences at the extremities of the PPTase genes. The primers labelled with an asterisk were used to check the in-frame deletion. The underlined nucleotides are *Nde*I and *Xba*I restriction sites.
