# Supplementary material for: A Single Sfp-Type Phosphopantetheinyl Transferase Plays a Major Role in the Biosynthesis of PKS and NRPS Derived Metabolites in Streptomyces ambofaciens ATCC23877
Source: PLoS One. 2014 Jan 31;9(1):e87607. doi: 10.1371/journal.pone.0087607 (PMC3909215; doi:10.1371/journal.pone.0087607)
Supplement: Text S1 — Mass spectrometric methods. (PDF) [file pone.0087607.s013.pdf]

Text S1. Mass spectrometric methods

Mass spectrometric analysis of stambomycins

Qualitative and semi-quantitative analyses of stambomycins were performed using a HPLC-MS system (ThermoFisher Scientific, San Jose, CA, USA) consisting in a binary solvent delivery pump connected to a photodiode array detector (PDA) and in an LTQ ion trap as mass analyzer (Linear Trap Quadrupole) equipped with an atmospheric pressure ionization interface operating in positive electrospray mode (ESI^+). Chromatographic separation was performed on a C18 Alltima reverse phase column (150 x 2.1mm, 5µm porosity – Grace/Alltech, Darmstadt, Germany) equipped with a C18 Alltima pre-column (7.5 x 2.1 mm, 5 µm porosity – Grace/Alltech) at 25 °C. Mobile phases were in A: water (trifluorocacetic acid 0.1%) and B: acetonitrile (trifluorocacetic acid 0.1%). Stambomycins were analyzed using isocratic elution at 5% of B for 5 min and followed by linear gradient from 5% to 100% of B in 30 min at a flow rate of 0.2 ml/min. Mass spectrometry operating parameters were: spray voltage was set at 5 kV; source gases were set for sheath gas, auxiliary gas and sweep gas at 30, 10 and 10 arbitrary units/min, respectively; capillary temperature was set at 275 °C; capillary voltage was set at 4 V; tube lens, split lens and front lens voltages were set at 155 V, -28 V and -6 V, respectively. The ion optics parameters were optimized by automatic tuning using a standard solution of stambomycins C/D (m/z=682, $[M+2H]^{2+}$ discharged parent ion) at 0.1g/l infused in mobile phase (A/B: 50/50) at a flow rate of 5µl/min. Full scan MS spectra were acquired from 50 to 1500 m/z and MS^2 fragmentations of corresponding $[M+2H]^{2+}$ stambomycin parent ions were carried out, as follows: MS^2 (682) for stambomycins C/D, MS^2 (689) for stambomycins A/B. Chromatographic conditions used give the following retention time (RT) for compounds of interest: stambomycins C/D, RT 20.00 min; stambomycins A/B, RT 20.60 min.

Mass spectrometry analysis of siderophores compounds

Qualitative and semi-quantitative analyses of siderophore compounds were performed using a HPLC-MS system (ThermoFisher Scientific, San Jose, CA, USA) consisting in a binary solvent delivery pump

connected to a photodiode array detector (PDA) and in an LTQ ion trap as mass analyzer (Linear Trap Quadrupole) equipped with an atmospheric pressure ionization interface operating in positive electrospray mode (ESI^+). Chromatographic separation was performed on a C18 Alltima reverse phase column (150 x 2.1mm, 5µm porosity – Grace/Alltech, Darmstadt, Germany) equipped with a C18 Alltima pre-column (7.5 x 2.1 mm, 5 µm porosity – Grace/Alltech) at 25 °C. Mobile phases were in A: water (formic acid 0.1%) and B: acetonitrile (formic acid 0.1%). Siderophores were analyzed using a linear gradient from 0% to 35% of B in 20 min at a flow rate of 0.2 ml/min. Mass spectrometry operating parameters were as follows: spray voltage was set at 5 kV; source gases were set for sheath gas, auxiliary gas and sweep gas at 30, 10 and 10 arbitrary units/min, respectively; capillary temperature was set at 275 °C; capillary voltage was set at 4 V; tube lens, split lens and front lens voltages were set at 155 V, -28 V and -6 V, respectively. The ion optics parameters were optimized by automatic tuning using a standard solution of stambomycin (m/z=682, $[M+2H]^{2+}$ discharged parent ion) at 0.1g/l infused in mobile phase (A/B: 50/50) at a flow rate of 5µl/min. Full scan MS spectra were acquired from 50 to 1500 m/z and MS^2 fragmentations of corresponding $[M+H]^+$ siderophore ions were carried out, as follows: MS^2 (566) for desferricoelichelin, MS^2 (619) for ferricoelichelin, MS^2 (561) for desferrioxamine B, MS^2 (614) for ferrioxamine B, MS^2 (601) for desferrioxamine E, MS^2 (654) for ferrioxamine E. Retention times: desferricoelichelin, RT 3.20 min; desferrioxamine B, RT 14.80 min; ferrioxamine B, RT 12.50 min; desferrioxamine E, RT 18.20 min; ferrioxamine E, RT 15.50 min.
